# Structures and implications of the C962R protein of African swine fever virus

**DOI:** 10.1093/nar/gkad677

**Published:** 2023-08-17

**Authors:** Zhiwei Shao, Shichen Su, Jie Yang, Weizhen Zhang, Yanqing Gao, Xin Zhao, Yixi Zhang, Qiyuan Shao, Chulei Cao, Huili Li, Hehua Liu, Jinru Zhang, Jinzhong Lin, Jinbiao Ma, Jianhua Gan

**Affiliations:** Shanghai Public Health Clinical Center, State Key Laboratory of Genetic Engineering, Collaborative Innovation Center of Genetics and Development, Department of Biochemistry and Biophysics, School of Life Sciences, Fudan University, Shanghai 200438, China; State Key Laboratory of Genetic Engineering, Collaborative Innovation Center of Genetics and Development, Department of Biochemistry and Biophysics, School of Life Sciences, Fudan University, Shanghai 200438, China; Shanghai Public Health Clinical Center, State Key Laboratory of Genetic Engineering, Collaborative Innovation Center of Genetics and Development, Department of Biochemistry and Biophysics, School of Life Sciences, Fudan University, Shanghai 200438, China; Shanghai Public Health Clinical Center, State Key Laboratory of Genetic Engineering, Collaborative Innovation Center of Genetics and Development, Department of Biochemistry and Biophysics, School of Life Sciences, Fudan University, Shanghai 200438, China; Shanghai Public Health Clinical Center, State Key Laboratory of Genetic Engineering, Collaborative Innovation Center of Genetics and Development, Department of Biochemistry and Biophysics, School of Life Sciences, Fudan University, Shanghai 200438, China; Shanghai Public Health Clinical Center, State Key Laboratory of Genetic Engineering, Collaborative Innovation Center of Genetics and Development, Department of Biochemistry and Biophysics, School of Life Sciences, Fudan University, Shanghai 200438, China; Shanghai Public Health Clinical Center, State Key Laboratory of Genetic Engineering, Collaborative Innovation Center of Genetics and Development, Department of Biochemistry and Biophysics, School of Life Sciences, Fudan University, Shanghai 200438, China; Shanghai Public Health Clinical Center, State Key Laboratory of Genetic Engineering, Collaborative Innovation Center of Genetics and Development, Department of Biochemistry and Biophysics, School of Life Sciences, Fudan University, Shanghai 200438, China; Shanghai Public Health Clinical Center, State Key Laboratory of Genetic Engineering, Collaborative Innovation Center of Genetics and Development, Department of Biochemistry and Biophysics, School of Life Sciences, Fudan University, Shanghai 200438, China; Shanghai Public Health Clinical Center, State Key Laboratory of Genetic Engineering, Collaborative Innovation Center of Genetics and Development, Department of Biochemistry and Biophysics, School of Life Sciences, Fudan University, Shanghai 200438, China; Shanghai Public Health Clinical Center, State Key Laboratory of Genetic Engineering, Collaborative Innovation Center of Genetics and Development, Department of Biochemistry and Biophysics, School of Life Sciences, Fudan University, Shanghai 200438, China; State Key Laboratory of Genetic Engineering, Collaborative Innovation Center of Genetics and Development, Department of Biochemistry and Biophysics, School of Life Sciences, Fudan University, Shanghai 200438, China; State Key Laboratory of Genetic Engineering, Collaborative Innovation Center of Genetics and Development, Department of Biochemistry and Biophysics, School of Life Sciences, Fudan University, Shanghai 200438, China; State Key Laboratory of Genetic Engineering, Collaborative Innovation Center of Genetics and Development, Department of Biochemistry and Biophysics, School of Life Sciences, Fudan University, Shanghai 200438, China; Shanghai Public Health Clinical Center, State Key Laboratory of Genetic Engineering, Collaborative Innovation Center of Genetics and Development, Department of Biochemistry and Biophysics, School of Life Sciences, Fudan University, Shanghai 200438, China

## Abstract

African swine fever virus (ASFV) is highly contagious and can cause lethal disease in pigs. Although it has been extensively studied in the past, no vaccine or other useful treatment against ASFV is available. The genome of ASFV encodes more than 170 proteins, but the structures and functions for the majority of the proteins remain elusive, which hindered our understanding on the life cycle of ASFV and the development of ASFV-specific inhibitors. Here, we report the structural and biochemical studies of the highly conserved C962R protein of ASFV, showing that C962R is a multidomain protein. The N-terminal AEP domain is responsible for the DNA polymerization activity, whereas the DNA unwinding activity is catalyzed by the central SF3 helicase domain. The middle PriCT2 and D5_N domains and the C-terminal Tail domain all contribute to the DNA unwinding activity of C962R. C962R preferentially works on forked DNA, and likely functions in Base-excision repair (BER) or other repair pathway in ASFV. Although it is not essential for the replication of ASFV, C962R can serve as a model and provide mechanistic insight into the replicative primase proteins from many other species, such as nitratiruptor phage NrS-1, vaccinia virus (VACV) and other viruses.

## Graphical Abstract

**Graphical Abstract:**
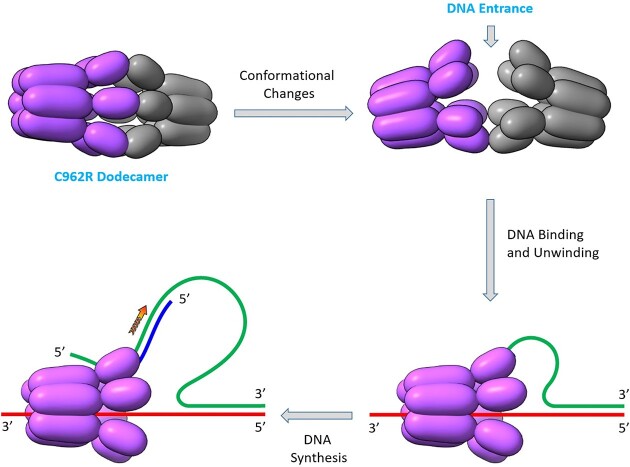

## INTRODUCTION

African swine fever virus (ASFV) is the etiological agent of African swine fever (ASF), which is contagious and highly lethal to domestic pigs and wild boars. ASF was first reported in Kenya in 1921 and restrained to Africa till the mid-1950s (1). Since then, ASF has been spread into many countries in South America, Europe and Asia, turned into a global threat to the swine industry (2–4). In 2018, ASF emerged in China (5), the world's largest pork producer, caused immediate pork shortage and huge economic losses. Although it has been extensively studied in the past, no useful ASFV vaccine or treatment has been developed (6). To date, ASF outbreaks are still controlled by massive killing and burying pigs on the infected farms.

ASFV is a double-stranded (ds) DNA virus; it belongs to the *Asfivirus* genus and is the only known member of the *Asfarviridae* family. As one of the most complex DNA viruses, the genome of ASFV varies between 170 and 193 kb and encodes >170 proteins that function in various stages of the ASFV life cycle (7,8). The structures of ASFV virion assembly pathway proteins have been reported by Rao and coworkers (9). Our group determined the crystal structures of *Asfv*AP, *Asfv*PolX and *Asfv*LIG, three key enzymes involved in ASFV DNA base-excision repair (BER) pathway; in addition to the catalytic mechanism, these structures also revealed many features unique to ASFV (10–12). Unlike the virion assembly or the BER pathway proteins, the structures of many other ASFV proteins have not been determined, which hinders our understanding on the detailed mechanisms of entry into host cells, suppression of host immune response, DNA replication, gene expression and translation of ASFV.

ASFV encodes a set of DNA replication proteins, such as replicative DNA polymerase (*Asfv*DNAP), DNA topoisomerase II (*Asfv*TopII) and proliferating cell nuclear antigen (*Asfv*PCNA) (13). The functional importance of *Asfv*DNAP and *Asfv*TopII has been well confirmed by *in vivo* studies, but the catalytic mechanism of ASFV DNA replication pathway is largely unclear, due to the lack of structural information. Here, we report two cryo-EM structures of C962R, showing that the full-length C962R adopts a ring-shaped structure. In addition to the AEP domain (Archaeo-eukaryotic primase domain) at the N-terminus, C962R also contains a PriCT2 domain, a D5_N domain and a SF3 helicase domain in the middle. The conformations of the PriCT2 and D5_N domains are conserved in the apo and the complexed structures, but binding of AMPPNP (analog of ATP) and single-stranded (ss) DNA leads to obvious conformational rearrangement of the helicase domain and the Tail domain, which locates at the C-terminus of C962R. The DNA synthesis activity is performed by the AEP domain, but the helicase domain can significantly enhance the catalytic activity of the protein. Besides ASFV, our studies also shed light on the replication and repair mechanisms of VACV and many other viruses.

## MATERIALS AND METHODS

### Plasmid construction, protein expression and purification

The gene containing the codon-optimized cDNA sequence of C962R (Supplementary Table S1) was purchased from Suzhou Genewiz Co., Ltd, China. The target fragment was recombined into the pET-28a-SUMO vector. The recombinant 6 × His-SUMO-C962R coding vector was utilized as the template during the plasmid constructions of truncated C962R proteins. Plasmids for full-length mutant proteins were constructed using a Homologous Recombination kit (ClonExpress® Ultra One Step Cloning Kit, Vazyme), the detailed sequences of the primers were listed in Supplementary Table S2. All plasmids were transformed into *E. coli* Rosetta DE3 competent cells for protein expression. Sequences of all plasmids were confirmed by DNA sequencing.

All C962R proteins were expressed and purified using the same procedures. Briefly, the frozen recombinant strains were revived in Lysogeny broth (LB) medium supplemented with 50 μg/ml kanamycin at 37°C overnight. Every 15 ml revived bacterium suspension was inoculated into 1 l LB medium and cultured at 37°C. Protein expression was induced at OD_600_ ≈1.0 by adding isopropyl β-D-1-thiogalacto-pyranoside at a final concentration of 0.5 mM. The induced cultures were then grown at 18°C for an additional 20 h. The cells were collected via centrifugation, resuspended in Buffer A (20 mM Tris pH 8.0, 500 mM NaCl, 25 mM imidazole) and lysed under high pressure. The supernatant was loaded onto a HisTrap™ HP column and washed with Buffer B (20 mM Tris pH 8.0, 2 M NaCl). The target protein was eluted via ÄKTA pure (Cytiva) using Buffer C (20 mM Tris pH 8.0, 500 mM NaCl, 500 mM imidazole). The proteins were treated with Ulp protease and dialyzed against Buffer D (20 mM Tris pH 8.0, 500 mM NaCl). The sample was re-loaded onto the HisTrap™ HP column. The target proteins were collected, concentrated and loaded onto a Superose 6 Increase 10/300 GL column (Cytiva) equilibrated with Gel Filtration Buffer (20 mM Tris pH 8.0, 500 mM NaCl, 2 mM DTT). All proteins were analyzed using a 15% SDS-PAGE gel, concentrated to 20 mg/ml by centrifugal concentrator (Millipore, Burlington, MA, USA) and stored at -80 °C until use.

### Size-exclusion chromatography and AUC analysis

To analyze the oligomerization state of the C962R protein, the samples were applied to size-exclusion Superose 6 Increase 10/300 GL column (Cytiva) equilibrated with Buffer E (20 mM Tris pH 8.0, 100 mM NaCl, 2 mM DTT). The sedimentation velocity (SV) of the collected protein was analyzed using a Beckman/Coulter XL-I analytical ultracentrifuge. A volume of 380 μl of C962R protein and 400 μl of matching Buffer E were injected into appropriate channels of 12 mm double sector aluminum epoxy cells with sapphire windows. Solutions were centrifuged at 30 000 rpm at 20°C in an An-60Ti rotor for 4 h. Scans were collected at 280 nm with 45 s elapsed between each scan. The buffer composition (density and viscosity) and protein partial specific volume (V-bar) were obtained using the program SEDNTERP. The SV data were analyzed using the program SEDFIT.

### SEC-MALS analysis

The averaged molecular weight (MW) of C962R was also determined by multi-angle light scattering coupled with size-exclusion chromatography (SEC-MALS). 100 μl C962R proteins were injected into a Superose 6 Increase 10/300 GL column equilibrated with SEC–MALS buffer (20 mM Tris pH 8.0, 100 mM NaCl) at a 0.5 ml/min flow rate overnight. Elution of the proteins was monitored by three online detectors: UV detector (1200 Infinity LC System, Agilent Technologies, USA), light scattering detectors (DAWN HELEOS II, Wyatt Technology, USA) and refractive index detector (Optilab T-rEX, Wyatt Technology). Data analysis and molecular weight calculations were performed using the ASTRA v6.1.5.22 software (Wyatt Technology).

### Negative stain EM

Proteins collected from the peak at 11.8 ml of the Superose 6 Increase 10/300 GL column were diluted to 40 μg/ml using Buffer E. 5 μl of proteins were applied to the glow-discharged 200 mesh carbon-coated copper grids (Beijing Zhongjingkeyi Technology). The samples were stained using 0.75% uranyl formate and air-dried. Data were collected on a Talos L 120C transmission electron microscope equipped with a 4k × 4k CETA CCD camera. Images were recorded at a nominal magnification of ×73 000, corresponding to a pixel size of 1.95Å.

### Cryo-EM sample preparation and data collection

All full-length C962R protein samples used for Cryo-EM analysis were dissolved in Buffer F (20 mM HEPES pH 7.5, 100 mM NaCl, 5 mM MgCl_2_, 2 mM DTT). For the Apo-form structure, the protein collected from the 11.8 ml peak of the Superose 6 Increase 10/300 GL column was concentrated to 1.2 mg/ml. For the complex structure, the protein was mixed with 32nt poly(T) DNA in Buffer G (20 mM HEPES pH 7.5, 100 mM NaCl, 5 mM MgCl_2_, 2 mM DTT) and incubated at room temperature for 1 h. The mixture was then injected to the Superose 6 Increase 10/300 GL column, the peak was collected and concentrated to 1.2 mg/ml. AMPPNP was added to a final concentration of 0.5 mM. To enhance the stability of the structure, the sample was crosslinked using bis(sulfosuccinimidyl)suberate (BS3; Thermo Fisher Scientific), which was freshly resuspended in DNase-free water. The mixture was incubated at 25°C for 15 min and quenched by adding Tris-HCl pH 8.0 buffer. The optimal BS3 concentration was 0.2mM. The cross-linked complex was centrifuged at 20 000 × g for 5 min at 4°C to remove the remaining aggregates.

An aliquot of 3.5 μl of sample was applied to a copper R 1.2/1.3 300 mesh grid (Quantifoil), which was freshly glow-discharged (inPELCO easiGlow^TM^ Glow Discharge Cleaning System) for 60 s at middle level after 2 min evacuation. The grids were blotted by a couple of 55 mm filter papers (Ted Pella) at 22°C and 100% humidity for 3 s, flash-frozen in liquid ethane using the FEI Vitrobot Mark IV. Cryo-EM data were collected on Titan Krios electron microscopes operated at 300 kV equipped with a Gatan K3 direct electron detector. All data were automatically recorded using EPU in super resolution mode and defocus values ranged from −1.5 μm to −2.0 μm.

### Electron microscopy data processing, model building and validation

For both the apo and complex structures, image processing was adopted in similar steps, dose-weighted and summed using MotionCor2 (14). The following steps were then processed in RELION (v.3.1) (15). The contrast transfer function parameters were estimated using CTFFIND4 (16). Approximately 2000 particles were manually picked and 2D-classified to generate initial templates. Based on the templates, more particles were then automatically picked from raw micrographs. After one round of reference-free 2D classification and several rounds of 3D classification, the initial 3D reference models were built by *ab initio* calculation in RELION. Particles with ordered density and clear structural features were selected for further 3D refinement. Local resolution distribution was evaluated using RELION. The detailed image processing of each dataset is provided in Supplementary Figures S2 and S5. The model of the C962R monomer was predicted by AlphaFold2 (17) and docked into EM 3D density maps using the program ChimeraX. Model adjustment was manually done in ChimeraX (18) (ISOLDE plug-in) (19) and COOT (20). The resulting models were refined against the EM map by PHENIX in real space with secondary structure and geometry restraints. The final models were validated in the PHENIX software package. The model statistics are summarized in Supplementary Table S3.

### Crystallization, data collection and structural refinement

The isolated AEP domain of C962R (aa 1–285 or aa 21–272) were purified and utilized in the crystallization screen. For the complex formation, the protein, magnesium ion and/or dCTP were mixed; the final protein, manganese ion and dCTP (if present) concentrations are 50 mg/ml, 5 mM and 20 mM, respectively. The initial crystallization conditions were identified by the sitting-drop vapor-diffusion method using commercial crystal screening kits at 18°C. The drop contained an equal volume (0.2 μl) of protein sample and reservoir solution and was equilibrated against 50 μl of reservoir solution in a 96-well format. The reservoir solutions are composed of 0.1 M Bis–Tris propane pH 8.5, 20% PEG 3350 and 0.2 M Sodium iodide for the apo-form AEP structure, and 0.1 M Bis-Tris propane pH 7.5, 25% PEG 3350 and 0.2 M Sodium citrate for the AEP-Mn^2+^ complex. For the AEP-dCTP-Mn^2+^ complex, the reservoir solution is composed of 0.1 M SPG buffer pH 8.0 and 25% PEG 1500.

All crystals were cryoprotected using their mother liquor supplemented with 25% glycerol and snap-frozen in liquid nitrogen. The diffraction data were collected at beamlines BL02U1 and BL10U2 at the Shanghai Synchrotron Radiation Facility (SSRF) and beamline BL18U1 of National Facility for Protein Science Shanghai (NFPS). Data processing was carried out using the XDS or HKL3000 (21) program. The data collection and processing statistics were summarized in Supplementary Table S4. The apo-AEP structure was solved by molecular replacement (MR) method using the model predicted by AlphaFold2 and the Phaser program (22) of the CCP4 suite (23). The AEP-Mn^2+^ and AEP-dCTP-Mn^2+^ complexes were solved by MR method using the apo-AEP structure as the search model. The resulting models were refined against the diffraction data using the Refmac5 program of the CCP4 suite. The 2*F*_o_ – *F*_c_ and *F*_o_ – *F*_c_ electron density maps were regularly calculated and used as guide for the building of water, ions and dCTP in COOT. The final refinement of all structures was performed using the phenix.refine program. The structural refinement statistics were also summarized in Supplementary Table S4.

### ATPase activity assays

ATPase activities were measured using Malachite Green Phosphate Detection Kit (Beyotime). The assays were performed in a 20 μl reaction mixture containing 20 mM Tris pH 8.0, 100 mM NaCl, 5 mM MgCl_2_, 2 mM DTT, 1 mM ATP, and 40 nM Wild-type (WT) or mutated C962R proteins. The mixtures were incubated at 37°C for 30 min. The reactions were quenched by adding 180 μl 200 mM EDTA. 70 μl of malachite green reagent was added to the mixture. After 30 min, the absorbance was measured at 630 nm. Reactions without C962R were performed, served as a background reading of inorganic phosphate, and subtracted from the experimental results. The concentration of the released inorganic phosphate was calculated based on the absorbance curve of phosphate standards.

### DNA unwinding assay

The Top and the Bottom strands of DNA-2 and DNA-3 (Supplementary Table S5) were mixed with a molar ratio of 1:1. The mixtures were heated at 95°C for 5 min, followed by slow cooling to room temperature. The annealed DNAs (50 nM) were then mixed with C962R protein in the buffer composed of 20 mM Tris pH 8.0, 100 mM NaCl, 5 mM MgCl_2_, 1 mM ATP and 2 mM DTT. To prevent the re-annealing of the unwound Top strand and Bottom strand, 5 μM of non-labeled Bottom strand DNA was also included in the reaction system. After incubation at 37°C for 60 min, the reaction was terminated by adding EDTA to a final concentration of 20 mM. The C962R protein was digested by 5 mg/ml Proteinase K at room temperature for 30 min. Samples were then loaded onto 10% TBE polyacrylamide gel for electrophoresis on ice. The gel was visualized using Typhoon FLA 9000.

### DNA polymerization assay

The Template strand and the FAM-labeled Primer strand of DNA-1 (Supplementary Table S5) were mixed with a molar ratio of 1:1 in Buffer E. The mixtures were heated at 95°C for 5 min, followed by slow cooling to room temperature. The annealed DNA (1 μM) was then mixed with the full-length C962R protein or the isolated AEP domain in buffer composed of 20 mM Tris pH 8.0, 100 mM NaCl, 5 mM MgCl_2_, 2.5 mM dNTPs and 2 mM DTT. The reaction mixtures were incubated at 37°C. At specific time points, 5 μl aliquots of the reaction were quenched with 20 μl Formamide loading buffer (90% formamide, 20 mM EDTA, 0.05% bromophenol blue, and 0.05% xylene blue) and boiled at 95°C for 5 min. Samples were loaded onto pre-warmed 18% urea sequencing gels and run for 3 h. The gel was visualized using Typhoon FLA 9000.

Like DNA-1, the Template strand and the FAM-labeled Primer strand of DNA-4 were also annealed in Buffer E. Instead of the primer strand, the complementary strand was annealed with the template strand of DNA-5 (Supplementary Table S5) by heating at 95°C and slowly cooling to room temperature. The annealed DNA-4 or DNA-5 (2 μM) were mixed with WT or K642A mutant of C962R (0.5 μM) in buffer composed of 20 mM Tris pH 8.0, 100 mM NaCl, 5 mM MnCl_2_, 0.5 mM dNTPs, 10 mM ATP and 2 mM DTT. In the case of DNA-5, 1 μM FAM-labeled Primer strand was also included in the reaction buffer. The reaction was performed at 37°C and quenched at 15, 30, 45 and 60 min by mixing 5 μl of reaction mixtures with 20 μl Formamide loading buffer and boiling at 95°C for 5 min. The samples were analyzed by 18% urea sequencing gels and visualized using Typhoon FLA 9000.

### Terminal deoxynucleotidyl transferase assay

The terminal deoxynucleotidyl transferase assay was carried out using FAM-labeled Primer strand of DNA-1 (Supplementary Table S5). The DNA (1 μM) was mixed with the full-length C962R protein (0.5 μM) or the isolated AEP domain (50 μM) in buffer composed of 20 mM Tris pH 8.0, 100 mM NaCl, 5 mM MgCl_2_, 2.5 mM dNTPs and 2 mM DTT. The calf thymus Tdt (Beyotime Biotechnology) was utilized as positive control with a final concentration of 0.5 U. The reaction mixture was incubated at 37°C. At specific time points, 5 μl aliquots of the reaction were quenched with 20 μl Formamide loading buffer (90% formamide, 20 mM EDTA, 0.05% bromophenol blue, and 0.05% xylene blue) and boiled at 95°C for 5 min. Samples were loaded onto pre-warmed 18% urea sequencing gels and run for 3 h. The gel was visualized using Typhoon FLA 9000.

## RESULTS

### Structural assembly of the full-length C962R protein

**Figure 1.**
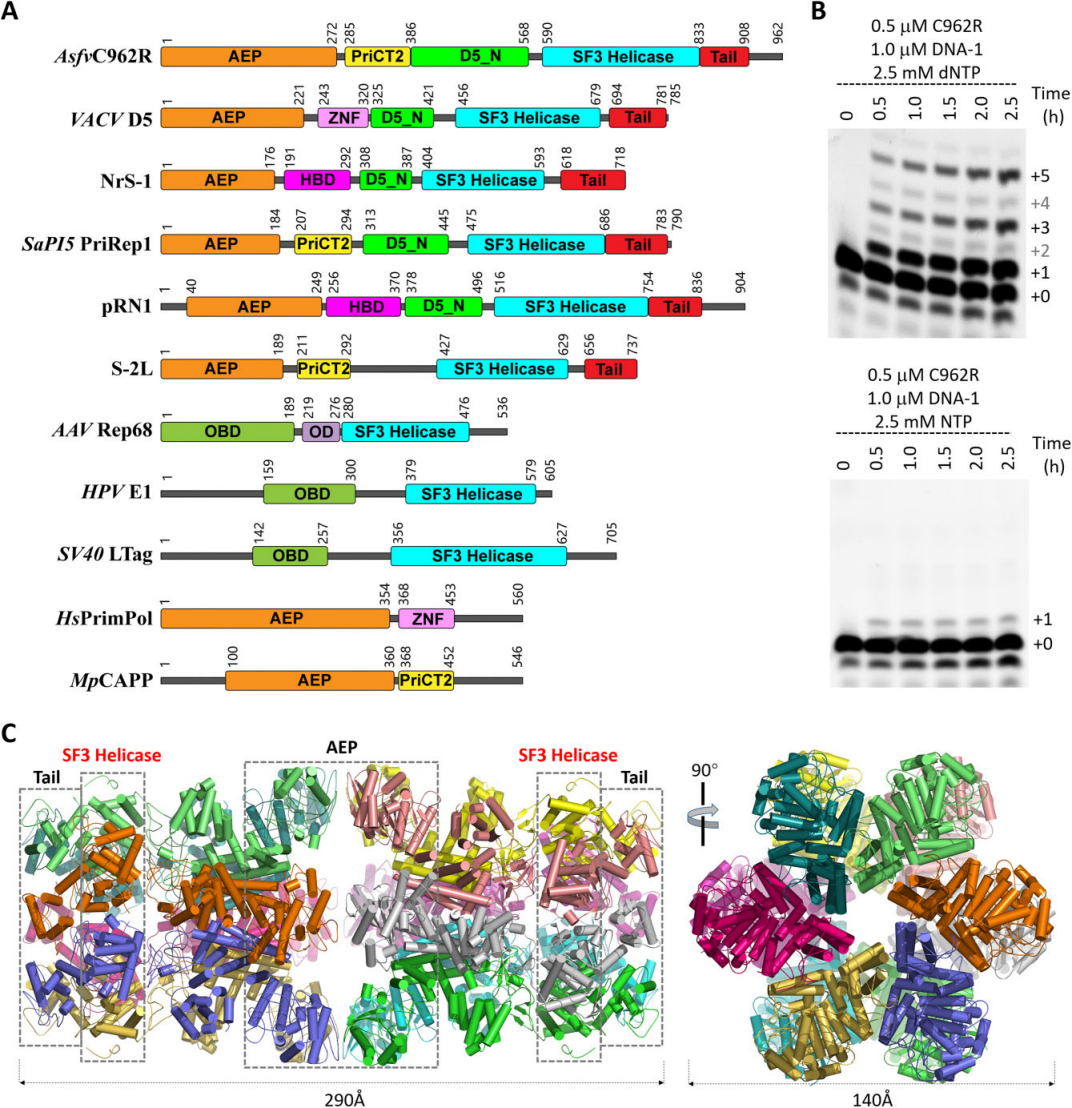
Primer extension and assembly of the full-length C962R protein. (**A**) Domain architectures of C962R and homologous proteins. (**B**) dNTP preference and primer extension activity of C962R. (**C**) Cryo-EM structure of the apo-form C962R protein.

C962R protein is encoded by the *C962R* gene (NCBI accession code: AJL34250.1); the mature protein is of 962 amino acids (aa) in length (Figure 1A). Sequence analysis suggested that C962R protein contains one AEP domain (aa 1–272) at the N-terminus, followed by one PriCT2 domain (aa 285–386), one D5_N domain (aa 387–568) and one SF3 helicase domain (aa 590–832) in the middle. No known domain was predicted for the C-terminal region (aa 833–962) of C962R. To better understand the function and catalytic mechanism of C962R, we expressed and purified the full-length protein. The protein was eluted from the Superose 6 Increase 10/300 GL column (Cytiva) at a volume of 11.8 ml (Supplementary Figure S1A). Comparison with the elution profile of standard proteins suggested that C962R forms oligomer in solution. To determine the exact oligomerization state of C962R, we performed AUC (Supplementary Figure S2) and SEC-MALS (Supplementary Figure S3) analysis, both assay results indicated that the full-length C962R mainly exists as dodecamer.

The presence of the AEP domain suggested that C962R likely possesses both *de novo* primer synthesis and primer-dependent polymerization activities. To experimentally confirm the catalytic abilities of C962R, we performed *in vitro* catalytic assays using the purified protein and DNA-1 (Supplementary Table S5), which are composed of a 17-nt template (5′-AGCTAAAGCGCATCCCG-3′) and 12-nt primer (5′-FAM-CGGGATGCGCTT-3′). As depicted in Figure 1B, C962R can only incorporate one NTP nucleotide to the 3′-end of the primer with extremely low efficiency. Although the dNTP incorporation efficiency of C962R is not very high, the full-length products can be observed on the gel. These observations indicated that C962R preferentially catalyzes the incorporation of dNTPs.

Upon the confirmation of the catalytic activity, we then performed structural studies for the full-length C962R protein. Images from the negative staining electron microscopy (EM) clearly showed that apo C962R protein forms highly organized oligomer (Supplementary Figure S4). To gain more insights into the assembly of C962R, the apo protein was subjected to 300 kV cryo-EM analysis (Supplementary Figure S5). The extracted particle images were divided into about forty classes by two-dimensional (2D) classification. Three-dimensional (3D) reconstruction and refinement further confirmed that C962R assembles into dodecamer (Figure 1C), which is consistent with the size-exclusion chromatographic profile, AUC and SEC-MALS analysis (Supplementary Figures S1–S3). C962R dodecamer is formed by two ring-shaped hexamers. The diameter of the outer ring is about 140 Å; and, the height of the C962R dodecamer is approximately 290 Å.

The C962R structure was refined up to an overall resolution of 3.67 Å (Supplementary Table S3). Although it cannot be predicted by the sequence, the structure (Supplementary Figure S6A) revealed a compact domain for the C-terminal Asp833-His910 region. The domain is of α/β fold in nature and was termed Tail domain hereafter. Dodecamerization of C962R is mediated by the AEP domains (Figure 1C), mainly through the hydrophobic interactions between the side chains of His120 and Tyr238 residues (Supplementary Figure S6B). No other dodecamerization interactions, such as salt-bridging interactions or hydrogen-binding (H-bond) interactions were observed in the structure.

### dNTP binding and incorporation by the AEP domain

**Figure 2.**
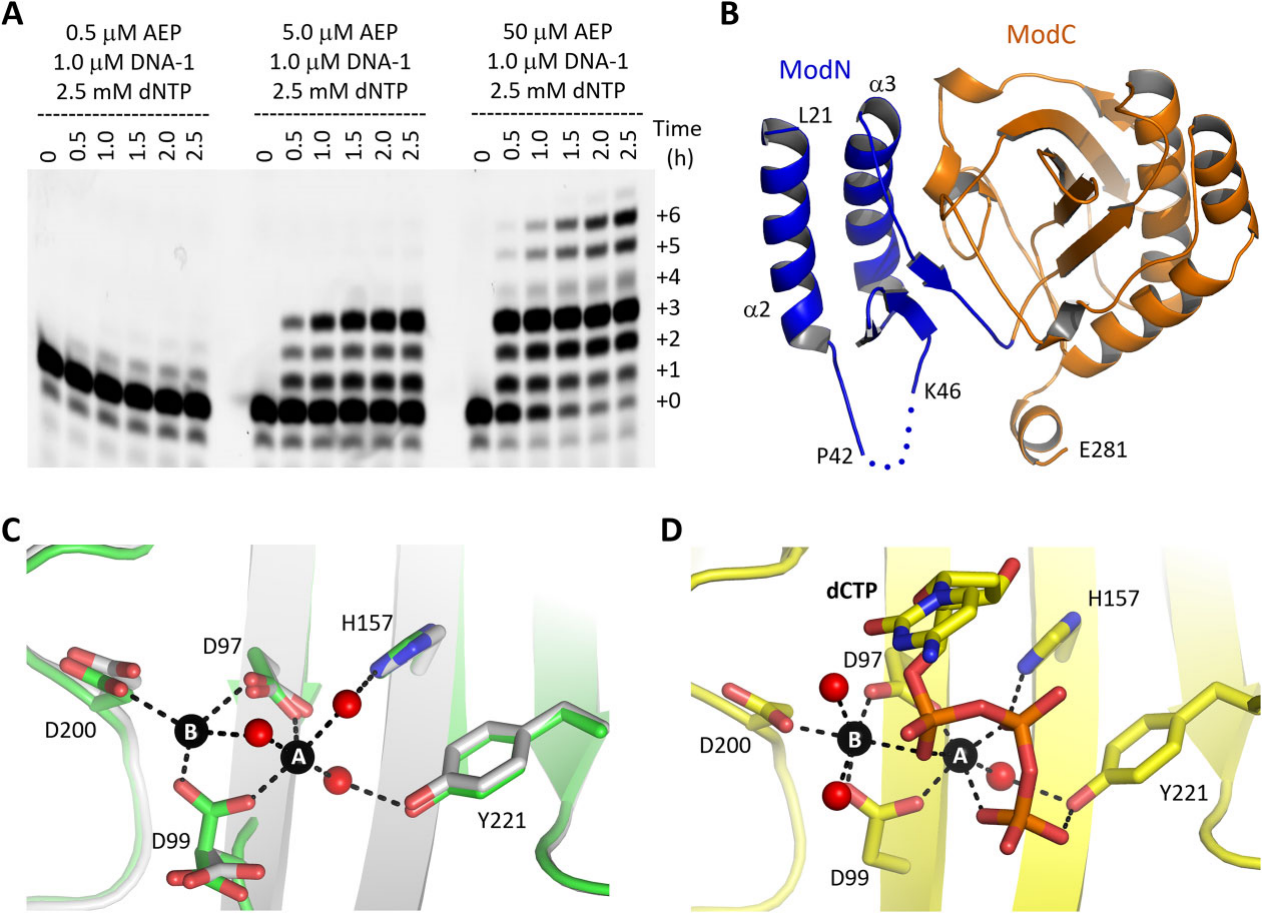
Structures and functional characterization of the AEP domain. (**A**) Primer extension assays catalyzed by the isolated AEP domain of C962R. (**B**) Overall folding of the AEP domain. The ModN and ModC subdomains are colored in blue and orange, respectively. (**C**) Mn^2+^ coordination and conformational changes observed in the apo- and the Mn^2+^-bound AEP structures. Mn^2+^ and the coordinating water molecules are shown as spheres in black and red, respectively. All C-atoms of the apo-form structure are colored in white, whereas in green in the complex structure. (**D**) Mn^2+^ coordination and dCTP binding in the AEP-dCTP-Mn^2+^ structure.

The full-length apo-form structure has shown that C962R is a multidomain protein (Supplementary Figure S6A). To clarify the function of individual domains, we constructed several C962R truncating variants. The proteins with either N- or C-termini truncated are very unstable, but the isolated AEP domain is stable and can be readily purified. Using the AEP domain and DNA-1, we performed *in vitro* DNA extension assays. Compared with the full-length protein (Figure 1B) at the same concentration (0.5 μM), the dNTP incorporation activity of the AEP domain is weaker (Figure 2A, left panel). Whereas, the AEP domain at higher concentrations (5 μM or 50 μM) could efficiently catalyze dNTP incorporation to the 3′-ends of the primers (Figure 2A, middle and right panels). No clear terminal deoxynucleotidyl transferase activity could be observed for either the full-length C962R protein or the isolated AEP domain (Supplementary Figure S7).

Our *in vitro* assays confirmed that the AEP domain is responsible for the DNA extension activity of C962R (Figure 2A). To reveal the underlying basis for dNTP binding and incorporation by the AEP domain, we performed crystallization studies. One apo-form structure was solved and refined up to 2.6-Å resolution (Supplementary Table S4), showing that the AEP domain is composed of two subdomains: ModN (aa 1–90) and ModC (aa 91–285). In the AEP domain structure (Figure 2B), the Met1-Tyr20 region is disordered. However, the region Asp10-Tyr20 is well ordered in the full-length C962R structure, folding into one α-helix (α1) and forming extensive hydrophobic interactions with the D5_N domains (Supplementary Figure S6C). The Ala273-Ala285 region forms one helix in the AEP domain structure, but is disordered in the full-length C962R structure.

Based on above structural observations, we constructed one new AEP variant (aa 21–272) and performed co-crystallization trials. One AEP-Mn^2+^ binary complex was solved at atomic resolution (Supplementary Table S4), revealed the detailed coordination of Mn^2+^ ions. As depicted in Figure 2C, the A-site Mn^2+^ coordinates with the side chains of Asp97 and Asp99 and three water molecules. Two of the water molecules form hydrogen bonding (H-bond) interactions with His157 or Tyr221, whereas the third water molecule coordinates with the B-site Mn^2+^. In addition to Asp97 and Asp99, the B-site Mn^2+^ also coordinates with the side chain of Asp200. Structural superposition showed that coordination of Mn^2+^ can dramatically change the conformation of Asp99 side chain (Figure 2C).

Using the new variant sample, we also solved one AEP-dCTP-Mn^2+^ ternary complex structure (Supplementary Table S4). As depicted in Figure 2D, the dCTP was captured at the active site of the protein, coordinating tightly with the Mn^2+^ ions. The A-site Mn^2+^ coordinates with the non-bridging oxygen atoms from all three phosphate groups, whereas the B-site Mn^2+^ only coordinates with the α-phosphate group of dCTP. Besides Mn^2+^ ions, the phosphate groups of dCTP also form H-bond interactions with the side chains of His157 and Tyr221. Structural comparison revealed that the orientations of the two Mn^2+^ ions are similar in the binary and ternary complex structures (Supplementary Figure S8A); the root mean square deviation (RMSD) values between the two complex structures and the apo-AEP structure are around 0.6–1.0 Å. Binding of dCTP has no obvious impacts on Asp97, Asp99, His157, Asp200 and Tyr221, but it leads to an approximately 100° rotation of the side chain of Tyr83.

The Leu21-Val37 region folds into one helix (α2) in the apo-form AEP structure, but it is disordered in the binary and ternary structures of the AEP domain (Supplementary Figure S8B). Disordering of N-terminal α-helices has also been observed in some other structures, such as α1 helix in the apo-form human (*Hs*) PrimPol structure. However, upon the binding of DNA, the α1 helix becomes well-ordered and stabilizes DNA from the major groove side (Supplementary Figure S8C). Superposition shows that the overall folding of the AEP domains is similar in our AEP-dNTP-Mn^2+^ structure and the *Hs*PrimPol-DNA-dATP complex (24). The conformations of the three acidic catalytic residues are also similar in the two structures. Likely, due to the lack of pairing with the template nucleotide, the nucleobase of dCTP in our structure is different from dATP in the *Hs*PrimPol complex; however, the triphosphate groups adopt similar conformations in the two structures (Supplementary Figure S8D).

In addition to *Hs*PrimPol, the AEP domain structures have also been reported for many other primase proteins, such as NrS-1 polymerase (25), *Cyanophage* S-2L PrimPol (26), and CRISPR-associated Primase-polymerase (CAPP) from *Marinitoga piezophila* (27). Although the sequence similarity between the AEP domains of C962R, NrS-1 polymerase, S-2L PrimPol and CAPP are very low (Supplementary Figure S9), the folding of the AEP domains are similar (Supplementary Figure S10). Conservation in catalytic residues, cation-coordination and dNTP binding (Supplementary Figures S8D and S10D) suggests that C962R may follow a conserved two-cation-assisted mechanism in dNTP incorporation as other primase family proteins.

### C962R possesses strong DNA unwinding activity

**Figure 3.**
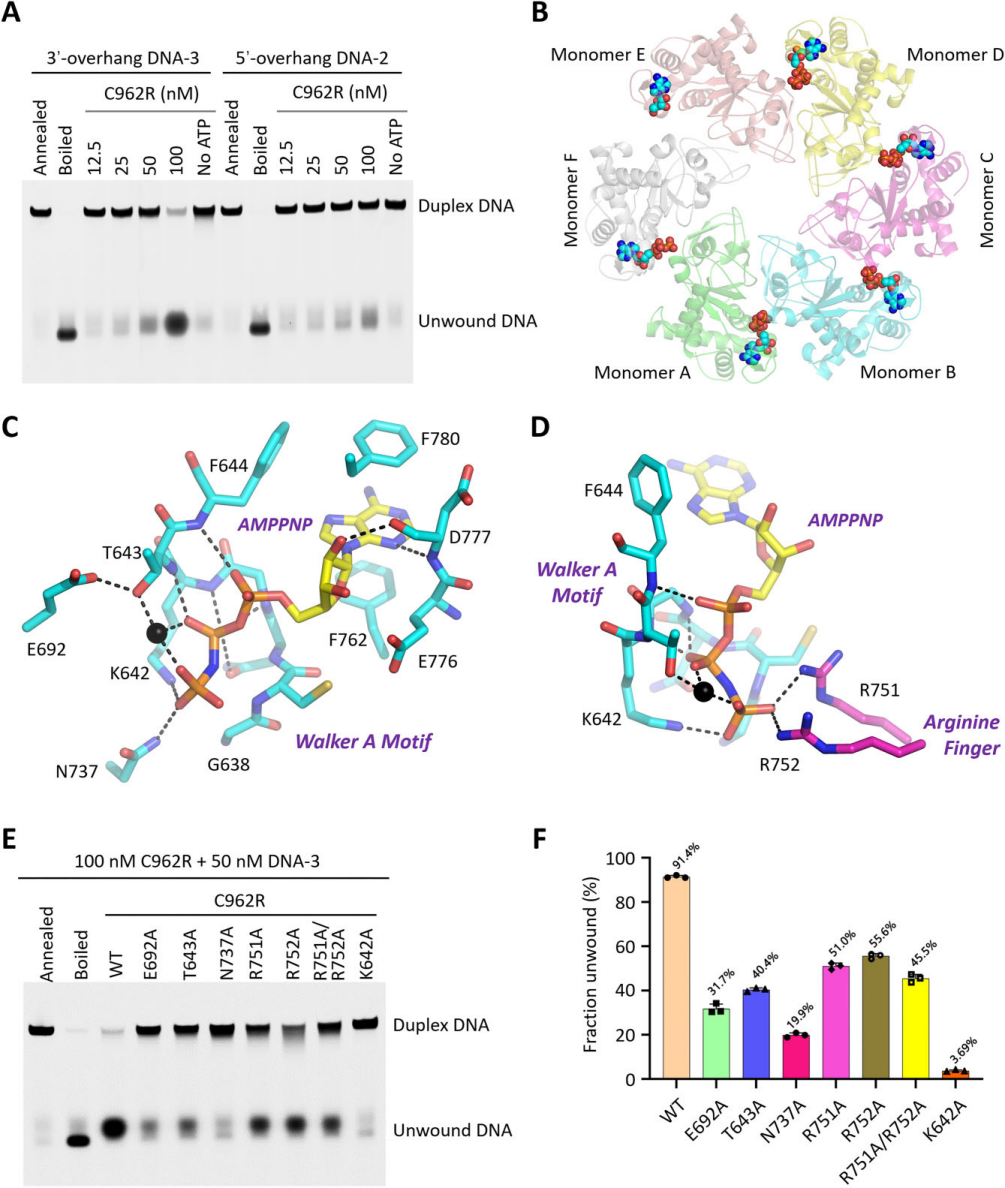
DNA unwinding and cofactor binding by the helicase domain of C962R. (**A**) *In vitro* DNA unwinding assays catalyzed by WT C962R. The concentration of C962R is 100 nM in the lane without ATP. (**B**) Conformations of AMPPNP and the helicase domains in the C962R-DNA-AMPPNP complex. AMPPNPs are shown as spheres. (**C, D**) The detailed interactions between AMPPNP and C962R. The Mg^2+^ is shown as black sphere. The C-atoms of Arg751 and Arg752 of the neighboring monomer are colored in magenta. (**E, F**) Comparison of *in vitro* DNA unwinding activities of WT C962R and mutants with AMPPNP-interacting residue mutated.

Helicase can be divided into various groups (28–31). The ring-shaped assembly (Figure 1C) suggested that the helicase domain of C962R may belong to the RecA-like group or the SF3 AAA+ group, which unwinds DNA duplex from the opposite directions. To confirm the helicase activity of C962R, we performed *in vitro* unwinding assays using 50 nM FAM-labelled DNAs (Supplementary Table S5). As depicted in Figure 3A, C962R has very low unwinding activity towards DNA duplex with 5′-overhang (DNA-2, Supplementary Table S5). However, >90% of 3′-overhang DNA substrates (DNA-3, Supplementary Table S5) can be unwound by C962R at a concentration of 100 nM. The 3′ to 5′ polarity of C962R helicase domain is similar to the classic SF3 AAA + helicases.

To reveal the detailed basis for DNA unwinding by C962R, we carried out cryo-EM study for C962R in the presence of both DNA and AMPPNP (Supplementary Figure S11). The 2D classification results showed that C962R assembled into dodecamer in the C962R-DNA-AMPPNP ternary complex (Supplementary Figure S12). However, different from the apo-form C962R structure (Figure 1C), the two C962R hexamers are tilted with respect to each other in the ternary complex structure. Since the tilting angle varies from one particle to another (Supplementary Figure S12A), we only modeled one C962R hexamer in the final structure (Supplementary Table S3). In the complex structure, six AMPPNP molecules were captured by the C962R hexamer (Figure 3B). The conformation of AMPPNP was stabilized by various types of interactions (Figure 3C, D). Besides the H-bond interaction with the main chain N atom of Asp777, the nucleobase of AMPPNP also forms hydrophobic stacking interactions with the side chains of Phe762 and Phe780. The 2′-OH group of AMPPNP sugar pucker forms H-bond with the main chain O atom of Asp777. The α and β phosphate groups of AMPPNP form four H-bond interactions with the main chain atoms of the Walker A motif (Gly638-Phe644); the γ phosphate group interacts with the side chain of Lys642 and Asn737 (Figure 3C). In the complex structure, we also observed one well-defined Mg^2+^ ion, which tightly coordinates with the side chain of Thr643 and the β and γ phosphate groups of AMPPNP. The side chain of Glu962 from the Walker B motif forms H-bond interaction with Thr643. In addition to above-mentioned interactions, the conformation of AMPPNP was further stabilized by its H-bond interactions with Arg751 and Arg752 from the neighboring C962R molecule (Figure 3D).

The ATP-interacting residues of C962R locate at the Walker A motif, Walker B motif, Sensor I, and Arginine-Finger, which are highly conserved in SF3 AAA+ group (Supplementary Figure S13). To investigate the functional importance of the ATP-interacting residues, we constructed several C962R mutants and performed *in vitro* DNA unwinding assays (Figure 3E-F). Compared with the wild-type (WT) protein, the DNA-3 unwinding activities of the T643A, E692A and N737A mutants are weaker. At a concentration of 100 nM, the T643A and E692A mutants unwound approximately 40% and 30% of the DNA substrates, respectively. Only about 20% DNA-3 were unwound by the N737A mutant. The DNA unwinding activities of the R751A, R752A and R751A/R752A mutants are higher than that of the T643A mutant, whereas they are all weaker than the WT protein. The most dramatic reduction was observed for the K642A mutant; under the same condition, the K642A mutant could only unwound 3.7% DNA-3, which is more than 20-fold weaker than the WT protein. In addition to DNA unwinding, we also compared the ATPase activities of WT and mutated C962R. As depicted in Supplementary Figure S14, WT C962R can efficiently hydrolyze ATP, whereas no clear ATP hydrolysis activity could be observed for the K642A mutant. Compared to WT C962R, the ATPase activities of the T643A, E692A, N737A, R751A, R752A and R751/752A mutants are significantly lower, indicating the functional importance of these ATP-interacting residues.

### Both D5_N and SF3 helicase domains participate in DNA binding and unwinding

**Figure 4.**
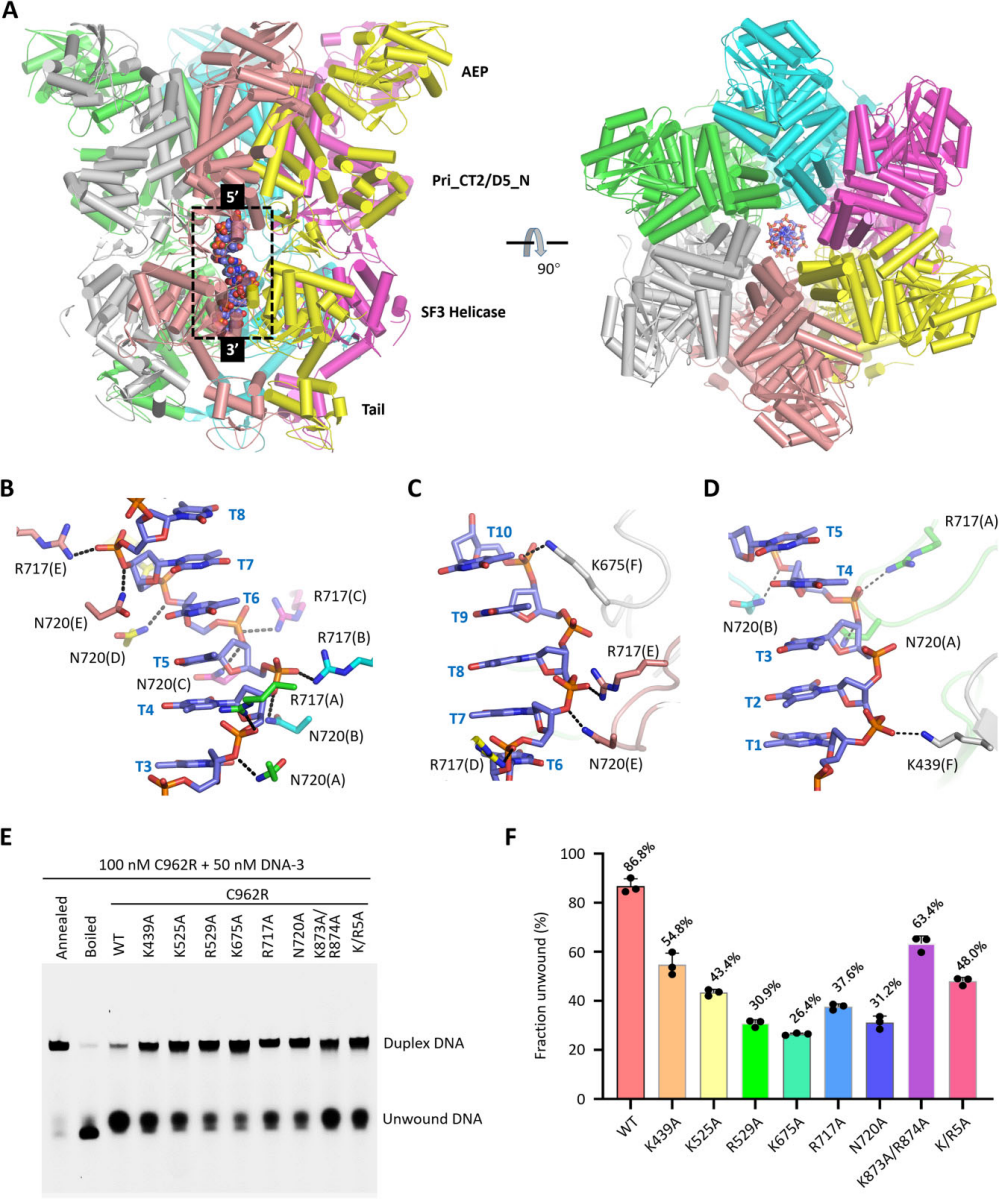
DNA binding by C962R. (**A**) The overall conformation of DNA in the C962R-DNA-AMPPNP complex. (**B–D**) The Detailed interactions between DNA and the SF3 helicase domain of C962R. The C-atoms of DNA are colored in light blue. The C-atoms of Arg717 and Asn720 of monomers A-F are colored differently. (**E, F**) Comparison of *in vitro* DNA unwinding activities of WT C962R and mutants with DNA-interacting residue mutated.

AMPPNP can mimic ATP in interacting with C962R, but it could not support the DNA unwinding and translocating activities. Therefore, instead of regular dsDNAs or dsDNAs with 3′-overhang, ssDNA was directly utilized in the cryo-EM study of the C962R-DNA-AMPPNP complex (Supplementary Table S3). The ssDNA is composed of 32 consecutive Thymidine nucleotides. Although some nucleotides were disordered, 10 Thymidine nucleotides (numbered T1 to T10) can be unambiguously modeled in the final structure. As depicted in Figure 4A, the DNA is located in the central channel of the C962R hexamer. The 5′- and 3′-ends of the DNA point towards the N- and C-termini of C962R, respectively.

The ssDNA adopts a B-form-like conformation in the complex structure. Although no stable interaction was observed for the nucleobases, the backbones of the DNA form extensive H-bond interactions with C962R, especially Arg717 and Asn720 of the helicase domains. As depicted in Figure 4B, Arg717 and Asn720 of C962R monomers A to E are spirally arranged. The side chains of Arg717 interact with the non-bridging OP1 atoms of T4-T8 nucleotides, respectively; Asn720 residues interact with the bridging O3’ atoms of T3-T7 nucleotides.

Instead of Arg717 or Asn720, the F monomer of C962R utilizes Lys675 and Lys439 in interacting with the DNA. Lys675 belongs to the SF3 helicase domain; it forms stable H-bond interaction with the phosphate backbone of T10 (Figure 4C). Lys439 forms H-bond interaction with the OP1 atom of T2 (Figure 4D). Lys439 locates at the middle of the D5_N domain, which assembled into a symmetric ring with an inner diameter of 21 Å (Supplementary Figure S15A). Although not directly involved in DNA binding in the complex structure, we noticed that the side chains of D5_N domain residues Lys525 and Arg529 all point towards the center of the ring (Supplementary Figure S15B). The D5_N domain contains three α-helices. Lys525 and Arg529 belong to the α3′ helix, which is roughly perpendicular to the α2′ helix. Like α3′, the α2′ helix also contains many positively charged residues, such as Lys505, Lys506, Lys509, Arg513 and Lys517 (Supplementary Figure S15C).

To investigate the potential function of aforementioned residues, we constructed several C962R mutants, including K439A, K525A, K529A, K675A, R717A, N720A and K/R5A, in which all the five positively charged residues of the α2′ helix are substituted by Ala residues. Using DNA-3, WT and mutated C962R proteins, we performed *in vitro* DNA unwinding assay. As depicted in Figure 4E, F, the DNA-3 unwinding activities of the K439A and K525A mutants are approximately 40–50% weaker than that of the WT protein. The DNA-3 unwinding activities of the R529A, K675A, R717A and N720A mutants are even weaker. Under the identical reaction condition, they can only unwind 26–38% of the DNA-3 substrates. The DNA-3 unwinding activity of the K/R5A mutant is comparable to that of the K439A mutant. Taken together, these observations suggested that Lys439, Lys525 and Lys529 of the D5_N domain and Lys675, Arg717 and Asn720 of the helicase domain all play important roles in DNA binding or unwinding by C962R.

### Complex formation leads to large conformational changes of C962R

Although only one hexamer was modeled in the final structure, C962R actually assembles into dodecamer in the C962R-DNA-AMPPNP complex (Supplementary Figure S12). Unlike the apo-form structure, the two C962R hexamers are tilted in the complex structure, created a large gap between the AEP domains. In combination with the *in vitro* DNA unwinding assay results (Figure 3A) and structural observations (Figure 4A), we believed that the gap likely serves as an entrance, which allows the DNA substrates to enter the central channel in a 3′-5′ direction (Supplementary Figure S12C).

**Figure 5.**
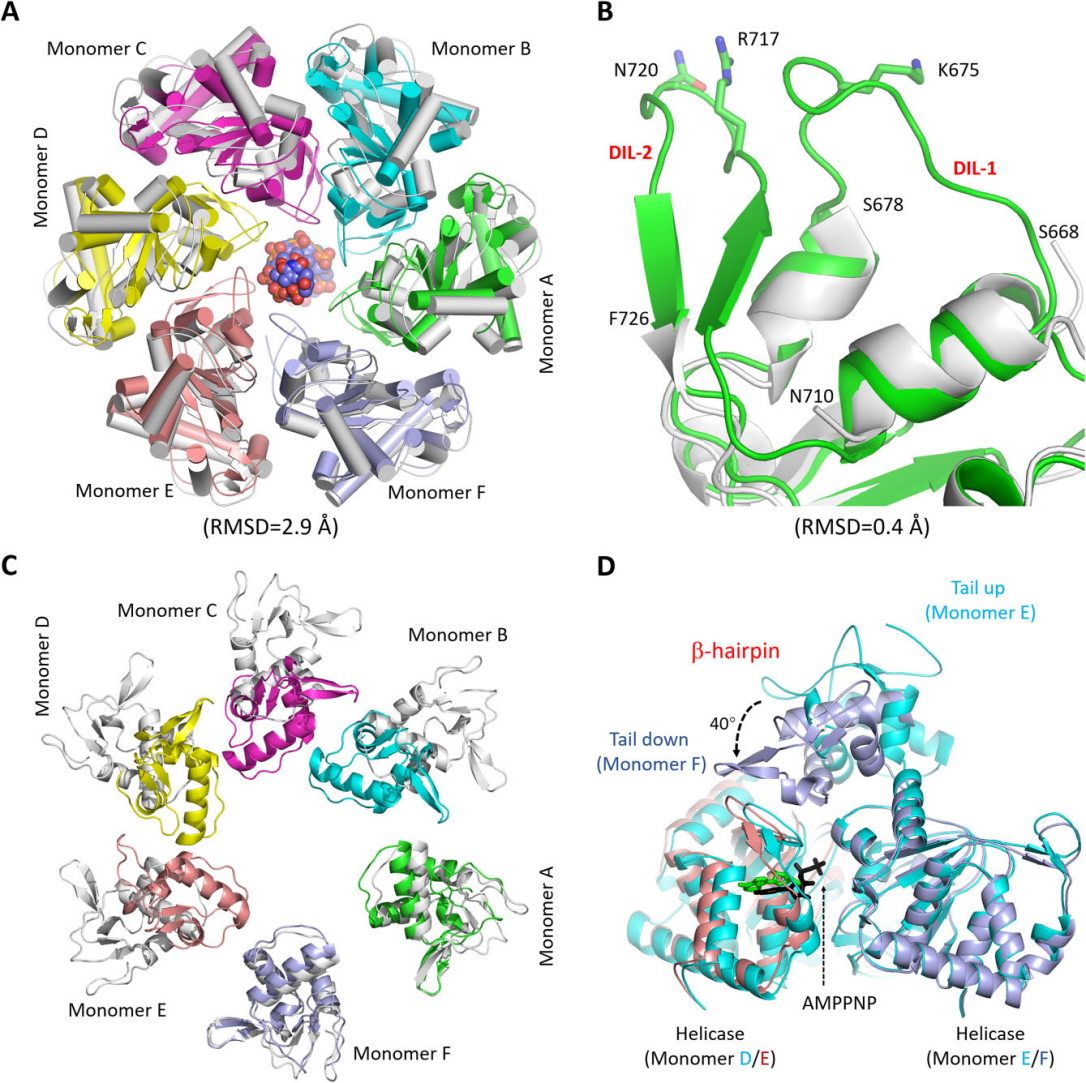
Conformational changes associated with DNA and cofactor binding by C962R. (**A**) Superposition of the SF3 helicase domains in the apo- and the complexed structures. The protein molecules and DNA are shown as cartoon and spheres, respectively. (**B**) Superposition showing the conformational changes of the two DIL loops of the SF3 helicase domain. (**C**) Conformational comparison of the Tail domains. The two structures were superposed based on the monomer F. (**D**) Superposition of the monomers E and F in the complex structure, showing the dramatically conformational difference of the Tail domains and their impacts on cofactor binding by the neighboring C962R monomers. In panels A–C, all C962R monomers are colored in white in the apo-form structure, but the six monomers are colored differently in the complex structure.

In addition to the relative orientations between the two hexamers, complex formation also leads to many conformational changes to the monomer and hexamer of C962R. As depicted in Supplementary Figure S16A, the central PriCT2 and D5_N domains of the apo and complexed C962R structures can superimpose well, but the relative orientations of the AEP, SF3 helicase and Tail domains are obviously different. Superposition showed that the overall folding and shape of the PriCT2/D5_N rings are similar in the two structures (Supplementary Figure S16B), supported by the low RMSD value (0.8 Å, based on 1725 pairs of Cα atoms). However, compared with the apo structure, the ring formed by the SF3 helicase domains is more condensed in the complex structure (Figure 5A). As aforementioned, the side chains of Lys675, Arg717 and Asn720 participate in DNA binding (Figure 4B-C). Lys675 locates at the tip of one long loop (aa 668–677); Arg717 and Asn720 belong to another loop (aa 716–723). The two loops are termed DNA-interacting loop (DIL) 1 and 2, respectively. Both DIL-1 and DIL-2 loops are well defined in the complex structures, whereas they are completely disordered in the apo structure (Figure 5B and Supplementary Figure S16C). In fact, the two β strands connected to DIL-2 are also disordered in the apo structure, further expanded the diameter of the central channel.

The SF3 helicase domains are symmetrically arranged in the apo C962R structure, whereas they adopt an asymmetric conformation in the complex structure. Structural superposition showed that the conformations of DIL-1 and DIL-2 are similar within the C, D and E monomers (Supplementary Figure S16D), but they are very different from those of the A and F monomers (Supplementary Figure S16E). Instead of Arg717 and Asn720, the different conformation allows Lys675 of the F monomer to participate in C962R and DNA interaction (Figure 4C). Compared with other monomers, the gap between the helicase domains of monomer E and F is significantly wider (Figure 3B). Unlike AMPPNP bound by other monomers, the triphosphate groups of AMPPNP bound by the E monomer are disordered. Other than the binding state, the partial AMPPNP better mimics ATP after the completion of hydrolysis.

Compared with the SF3 helicase domain, the Tail domain undergoes more dramatically conformational changes during complex formation. In the apo structure, the six Tail domains are symmetrically arranged along the central axis (Supplementary Figure S17A); the diameter of the inner ring is approximately 50 Å. In the complex structure, the Tail domains are asymmetrically arranged (Supplementary Figure S17B). Superposition showed that the overall folding and relative orientations of the Tail domains of the A and F monomers are similar in the two structures, but the Tail domains of the other four monomers are all shifted toward the A and F monomers in the complex structure (Figure 5C). With respect to the SF3 helicase domains, the Tail domains in the complex showed two different orientations: the Down orientation (for monomers A and F) and the Up orientation (for monomers B, C, D and E). Compared with monomer E, the Tail domain of monomer F is anti-clockwisely rotated about 40° (Figure 5D). Likely, to avoid clash with the rigid β-hairpin of the F monomer Tail domain, the helicase domain of monomer E is shifted, which may lead to the wider gap between the helicase domains of monomers E and F (Figures 3B and 5D).

No matter the main body (aa 833–910) is up-oriented or down-oriented, the conformation of one loop (aa 935–950) of the Tail domain is conserved (Supplementary Figure S17C, D). Via the side chains of Trp942, Trp943 and Trp945, the loop forms extensive hydrophobic interactions with the SF3 helicase domain in both the apo and the complex structures. The loop is termed helicase domain anchoring loop (HDAL) hereafter. To investigate the functional importance of the Tail domain, we constructed two C962R variants with the Tail domain or the HDAL loop deleted. Unlike the WT protein, the two variants are very unstable and could not be purified. We then did further structural analysis and found that the Tail domain contains two positively charged residues, Lys873 and Arg874. Similar to Lys527 and Arg529 of the D5_N domain, the side chains of Lys873 and Arg874 also point toward the central channel of the C962R hexamer (Supplementary Figure S17E). The K873A/R874A mutant of C962R can be readily purified. Compared with the WT protein, the K873A/R874A mutant showed weaker DNA-3 unwinding activity in the *in vitro* assay (Figure 4E).

### The SF3 helicase domain can enhance the polymerization activity of C962R

**Figure 6.**
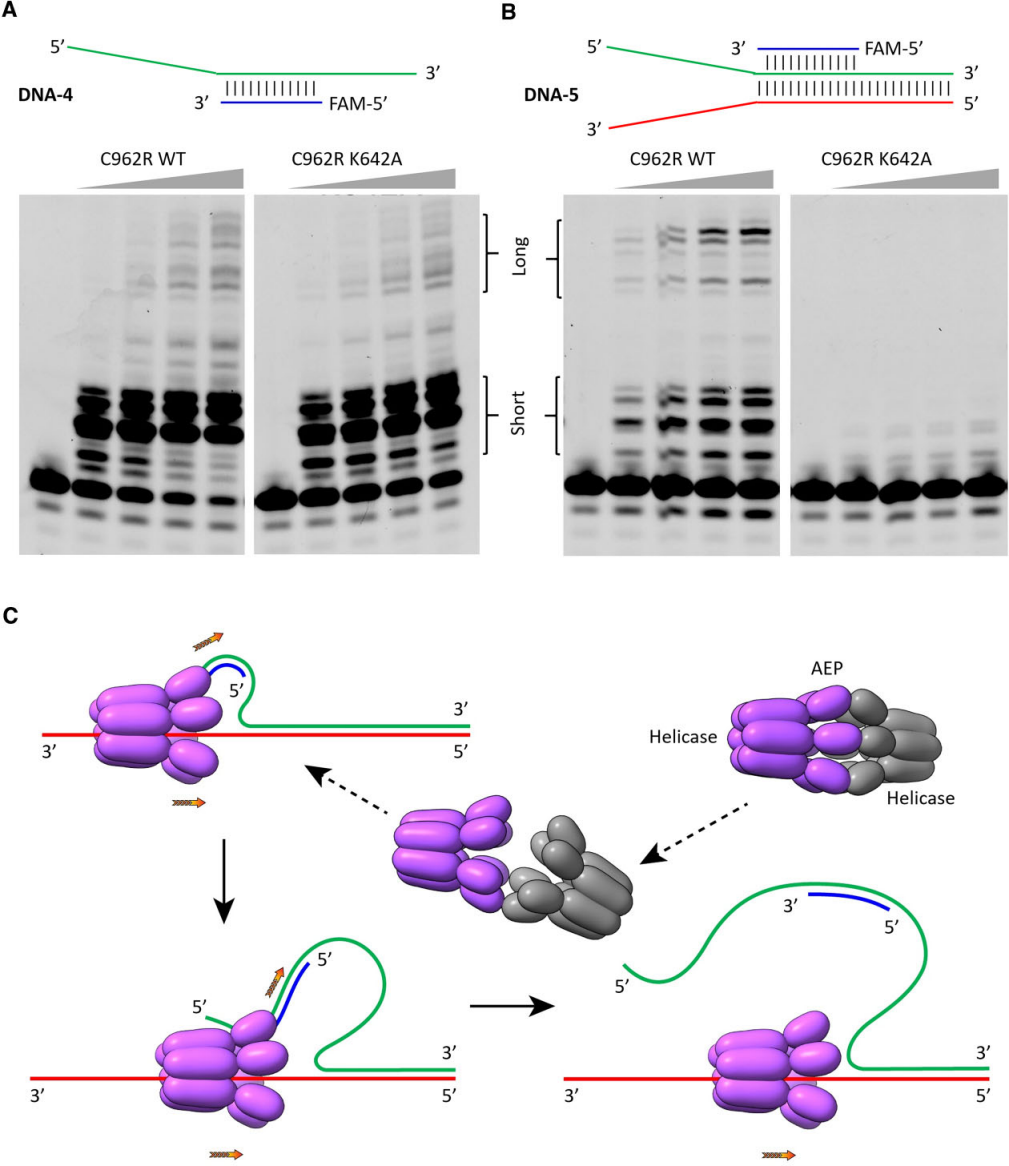
Correlation between DNA unwinding and polymerization activities of C962R. (**A, B**) *In vitro* DNA extension assays catalyzed by the WT C962R protein and the K642A mutant. The detailed sequences of the DNA substrates are listed in Supplementary Table S5. (**C**) One proposed model for the potential function of C962R.

As demonstrated above, C962R possesses both DNA polymerization and DNA unwinding activities, which are catalyzed by the AEP domain (Figure 2) and the SF3 helicase domain (Figure 3), respectively. The helicase domain preferentially unwinds DNA with 3′-overhag. The 3′-end of the DNA bound and unwound by the helicase domain points toward the C-terminal Tail domain, which is distant from the AEP domain (Figure 4A). The orientation of the unwound DNA suggested that it cannot serve as a template for the AEP-catalyzed DNA polymerization reaction. To investigate whether the DNA unwinding and polymerization activities of C962R are correlated, we synthesized two new DNAs: DNA-4 and DNA-5 (Supplementary Table S5). DNA-4 is composed of one 59-nt template strand and one 12-nt 5′-FAM-labeled primer strand, which pairs with the 22–33 nucleotides of the template. Using DNA-4 as substrate, we performed *in vitro* DNA polymerization assays (Figure 6A). Although not as efficient as the WT protein, the helicase-dead mutant K642A can catalyze the extension of the primer strand, suggesting that the helicase activity is not essential for C962R-catalyzed DNA-4 extension.

DNA-5 is composed of three strands: one template strand, one primer strand and one complementary strand. The sequences of the template and primer strands are identical to that of DNA-4. The complementary strand contains 59 nucleotides. The template and complementary strands can form 38 base pairs at their 3′ and 5′ ends, respectively; the resulting structure well mimics the forked DNA generated during DNA replication. Like DNA-4, we also performed *in vitro* DNA polymerization assays using DNA-5. As depicted in Figure 6B, the K642A mutant has no obvious catalytic activity toward DNA-5. In contrast, the WT protein can catalyze the primer extension of DNA-5. Compared with DNA-4 (Figure 6A), the yield for the short products is relatively lower, but the yield for the long products is higher for DNA-5. These observations suggested that the helicase activity is essential for the unwinding of the forked DNA, which in turn enhances the catalytic efficiency and processivity of C962R.

## DISCUSSION

In summary, we performed extensive structural and biochemical studies of C962R, showing that C962R possesses both DNA polymerization and DNA unwinding activities. C962R adopts a ring-shaped conformation, the N-terminal AEP domain is responsible for the DNA polymerization activity (Figure 2). Comparison of the AEP domain structures of C962R and homologous proteins (Supplementary Figures S8D and S10D) indicated that they follow one conserved two-cation-assisted mechanism in catalysis. The DNA unwinding activity is performed by the central SF3 helicase domain. Both structures and *in vitro* assay results (Figures 3 and 4) indicated that the helicase domain of C962R belongs to the SF3 AAA + superfamily, which unwinds DNA with a 3′-5′ polarity; other domains including the middle PriCT2 and D5_N domains and the C-terminal Tail domain all contribute to the DNA unwinding activity of C962R (Figure 4E, F). The DNA unwinding and polymerization activities of C962R are correlated; compared with the helicase-dead K642A mutant, the WT protein showed much higher DNA polymerization activity and processivity toward forked DNAs (Figure 6A, B).

C962R is encoded by the *C962R* gene. Although it shows some divergence in the E75 isolate and the South Africa isolates (RSA_2_2008 and SPEC_57_1985), the sequence of C962R is conserved in the highly virulent field isolate Georgia2007 (ASFV-G). Different from the PrimPol proteins in many other species, C962R could not be detected in the proteome of ASFV viral particles, which is critical for a DNA replication component. The *C962R* gene is transcribed as a late gene in ASFV, its transcription could only be detected 6 hours after infection. A recent study further showed that the C962R protein is non-essential and not strictly required for ASFV virulence in swine (32). However, careful analysis of the reported data showed that deletion of the *C962R* gene can slow down the evolution of mortality and body temperature of the infected swine.

ASFV is a very complex virus. In addition to *C962R*, the gene *F1055L* of ASFV also codes for one protein containing both AEP and helicase domains. Instead of SF3 AAA + group, the helicase domain of F1055L belongs to the SF2 group. In principle, F1055L could replace the function of C962R, maintaining the virulence of the ASFV-G-ΔC962R strain. However, no strain with both *C962R* and *F1055L* genes deleted has been reported; and, the structure and function of F1055L remain to be experimentally verified. Based on previous reports and our studies, we proposed on potential function for C962R. Instead of DNA replication, C962R likely works on the forked DNA generated during the DNA repair pathway, such as the base excision repair pathway. A plausible working model of C962R is shown in Figure 6C. In the absence of DNA, C962R exists as a dodecamer with the AEP domains located at the interface of the two hexamers. The weak hydrophobic interactions between the AEP domains allow the two hexamers to be easily tilted up, creating an entrance for the 3′-overhang of the forked DNA. Once the 3′-overhang enters the central channel, it will be bound and translocated by the SF3 helicase domain, releasing more nucleotides from the 5′-overhang region. Unlike the apo-form protein, the conformations of the AEP domain are very flexible in the complex, allowing the AEP domain to bind and move along the 5′-overhang and catalyze the priming of the DNA. Due to the opposite directions of unwinding and priming, the C962R-bound DNA forms a circular-like structure, which is similar to a replication bubble. The increasing tension will stop the priming and release the DNA from the C962R protein. It is of note that C962R exists as a dimer of two hexamers in the complex structure (Supplementary Figure S12). As an alternative to the mechanism in Figure 6C, both hexameric rings of C962R could bind and pump DNA to the interface between them, leading to the unwinding of the DNA.

Besides ASFV, SF3 AAA+ superfamily helicases also are present in many other DNA and RNA viruses, including simian virus 40 (SV40) (33,34), human papilloma virus (HPV) (35–37), adeno-associated virus (AAV) (38) and VACV (39). The large T antigen (LTag) from SV40 and the E1 protein from HPV (36) are the two well-known members of the SF3 AAA + helicases; they function in various stages of the viral replication cycle, from origin recognition to origin melting and unwinding. The Rep protein of AAV and the D5 protein of VACV also belong to the SF3 AAA+ superfamily. Although the overall sequence similarities are very lower, C962R shares some conserved features with other SF3 helicases (Supplementary Figure S13), such as the Walker A motif, the Walker B motif, DNA Sensor and the Arginine Finger, which carry the key residues Lys642 and Thr643, Glu692, Asn737 and Arg751 and Arg752, respectively. Like C962R, mutation of these key residues could significantly impair the function of other SF3 helicases (40).

The two DIL loops are largely disordered in the apo-form structure of C962R, whereas they form extensive interactions and drive the translocation of the DNA in the complex structure (Figure 5A, B). Similar phenomenon has also been observed for the Rep proteins from the Staphylococcus aureus pathogenicity islands (SaPIs) (41), the gp4 protein in bacteriophage T7 (42), and the E1 protein from HPV. Structural superposition showed that the arrangements of the helicase domains are similar in the DNA-bound C962R and E1 structures (36). In fact, the conformations of the DIL loops and the bound DNAs are also very similar in the two structures (Supplementary Figure S18A). The DNA-bound structures are still not available for NrS-1 polymerase, VACV D5, and many other SF3 helicase proteins. As depicted in Supplementary Figure S18B, NrS-1 polymerase (40) and VACV D5 (39) share very similar folding in their helicase domains with C962R, suggesting that they may follow one conserved mechanism in DNA binding and unwinding.

The domain architecture of C962R is similar to that of *SaPI5*PriRep1 (Figure 1A). In addition to AEP and SF3 helicase domains, C962R and *SaPI5*PriRep1 also contain one Tail domain at their C-termini. Tail domains are also present at the C-termini of VACV D5, NrS-1 polymerase, pRN1, and S-2L. However, the overall folding of these Tail domains are different from that of C962R. Instead, the folding of C962R Tail domain is similar to the Origin-binding domain (OBD) of phage P4 gpalpha protein (Supplementary Figure S19A) (43) and the DNA-binding domain (DBD) of the eukaryotic transcriptional activator Rfx1 (Supplementary Figure S19B) (44), which all belong to the winged helix protein family. As indicated by the mutagenesis and *in vitro* assays, the Tail domain plays certain role in DNA unwinding by C962R (Figure 4F). The sequence similarities between C962R Tail domain and the gpalpha and Rfx1 proteins are very low (Supplementary Figure S19C). It is unclear whether C962R Tail domain is involved in direct DNA binding, but its conformational changes likely affect ATP binding to the ATP-binding pockets between the helicase domains (Figure 5C, D).

Like C962R, the D5 protein of VACV also contains both the AEP domain and the SF3 helicase domains (Figure 1A). D5 has been suggested to play important role in the initiation of DNA replication or lagging strand DNA synthesis in VACV (45). As revealed by the Dali search program (46), the polymerase of NrS-1 also possesses similar domain architecture. The polymerase is critical for the replication of NrS-1 (47) and the C-terminal helicase domain can increase the polymerization activity of the N-terminal AEP domain (40). The PrimPol proteins contain a group of enzymes, which has been discovered widely spread in bacteria, archaeal and viruses (48). In addition to DNA replication, the dual DNA priming and extension activities allow PrimPol proteins to participate in various DNA damage repair processes (49). For example, human PrimPol protein (encoded by the CCDC111 gene) is required for both ultraviolet light-damaged DNA repair and oxidative lesion-containing DNA repair (50–52). The structures of many AEP domains or SF3 helicase domains have been reported, such as the helicase domain of VACV D5 (39), the AEP domain (25,53) and the helicase domain (40) of NrS-1 polymerase. However, the structures of the full-length D5 or NrS-1 polymerase proteins are unavailable. Our C962R–DNA complex represents the only DNA-bound structure of proteins containing both AEP and SF3 helicase domains. In many species, DNA unwinding and priming processes are performed by separated helicase and PrimPol proteins. Fusion of the two domains in one single chain may allow the protein to function more efficiently.

C962R is non-essential for ASFV replication, but it can serve as an excellent mode for the replicative PrimPol proteins, providing mechanistic insight into DNA replication in NrS-1, VACV and other viruses (54,55). Considered its high conservation in ASFV, it is worthy to further investigate the biological function of C962R, such as its involvement in DNA base-excision repair pathway, interactions with *Asfv*PCNA and the replicative DNA polymerase. The unique features of these replicative proteins will serve as ideal targets for small molecule design, which could help combat ASFV virus in the future.

## Supplementary Material

gkad677_Supplemental_File

## Data Availability

Structural factors and coordinates have been deposited in the Protein Data Bank under accession codes 8IQB, 8IQC and 8IQD for the AEP domain, the AEP-Mn^2+^ binary complex and the AEP-dCTP-Mn^2+^ ternary complex, respectively. Structural coordinates and cryo-EM maps have been deposited in the Protein Data Bank and Electron Microscopy Data Bank under accession codes 8IQH (EMDB-35670) and 8IQI (EMDB-35671) for the full-length apo-form C962R structure and the C962R-DNA-AMPPNP complex, respectively.
