# Supplementary material for: Structures and implications of the C962R protein of African swine fever virus
Source: Nucleic Acids Res. 2023 Aug 17;51(17):9475–90. doi: 10.1093/nar/gkad677 (PMC10516667; doi:10.1093/nar/gkad677)
Supplement: gkad677_Supplemental_File [file gkad677_supplemental_file.pdf]

Supplementary Information

For

Structures and implications of the C962R protein of African swine fever virus

Zhiwei Shao[1], Shichen Su[2], Jie Yang[1], Weizhen Zhang[1], Yanqing Gao[1], Xin Zhao[1], Yixi Zhang[1], Qiyuan Shao[1], Chulei Cao[1], Huili Li[1], Hehua Liu[1], Jinru Zhang[2], Jinzhong Lin[2], Jinbiao Ma[2], Jianhua Gan[1,*]

A

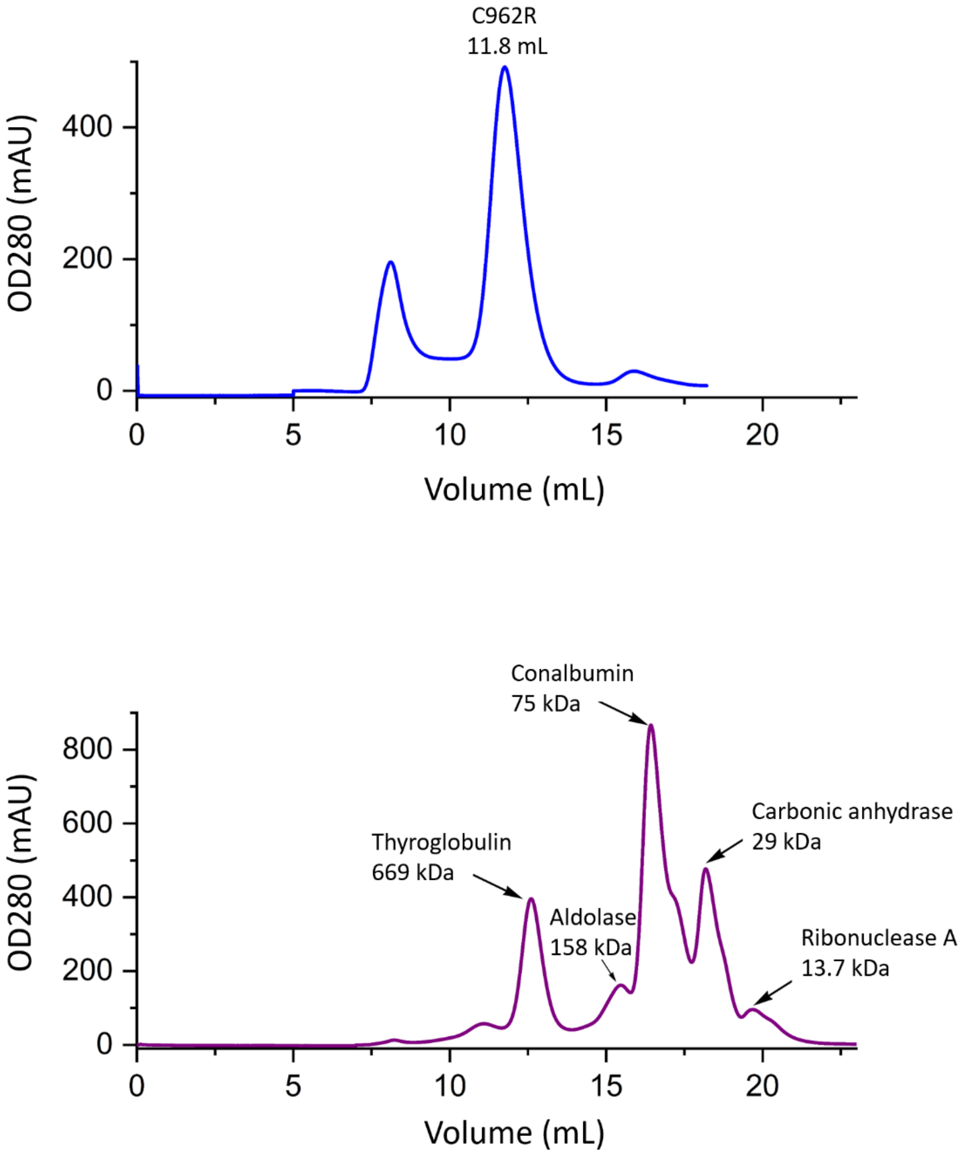

B

Fig. S1: Elusion profile of (A) the full-length C962R protein and (B) the standard proteins on the Superose 6 Increase 10/300 GL column.

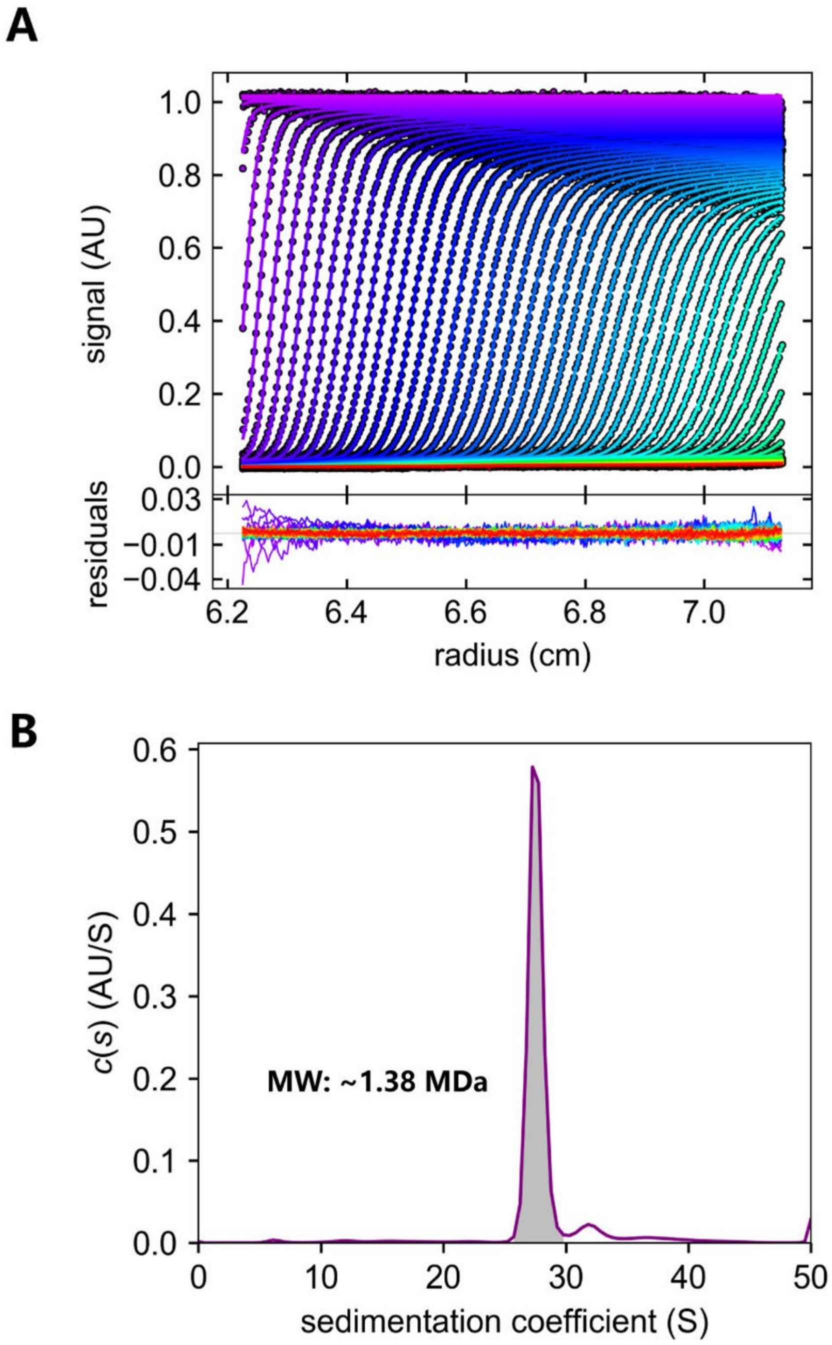

Fig. S2: Verification of the oligomerization state of the full-length C962R protein by analytical ultracentrifugation. (A) Representative interference scans of C962R monitored during sedimentation velocity analytical ultracentrifugation and residuals from fitting the data to a continuous c(s) distribution model as described in the methods. **(B)** c(S) distributions from SV runs for C962R.

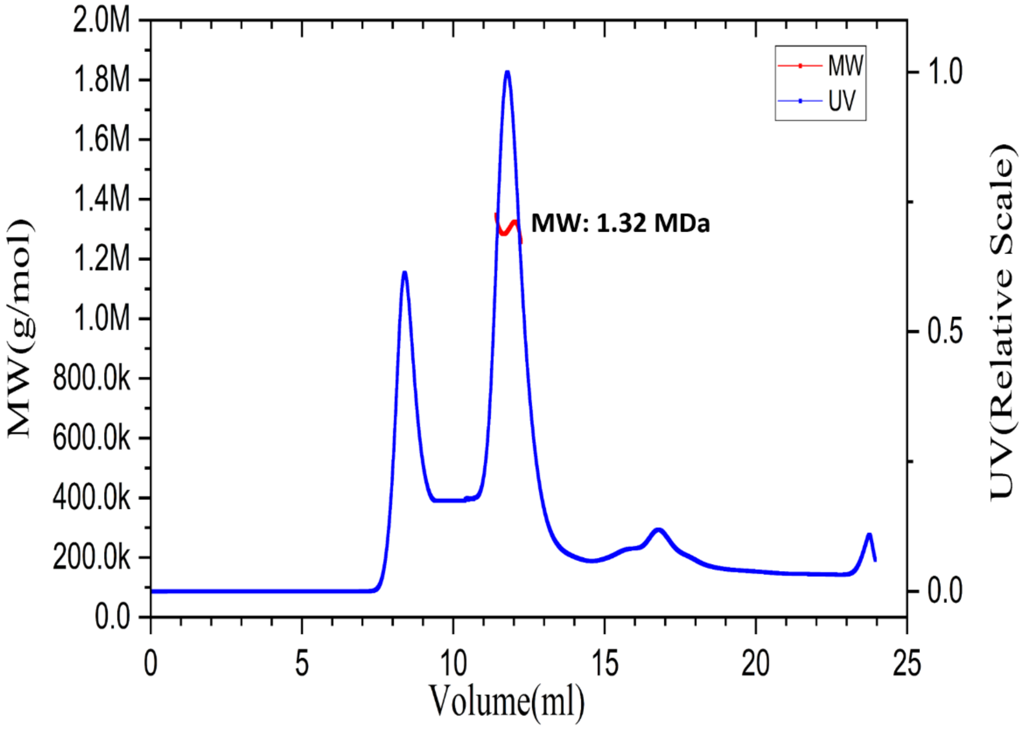

Fig. S3: Verification of the oligomerization state of the full-length C962R protein by SEC-MALS. SEC–MALS trace of C962R, the protein samples were eluted at 0.5 mL/min from a Superose 6 Increase 10/300 GL column (Cytiva) column. The right y axis is relative UV intensity value measuered by Agilent LC UV detector (Corresponds to the blue line), whereas the left y axis which is molecular mass determined by static MALS as described in experimental method (Corresponding to the red line).

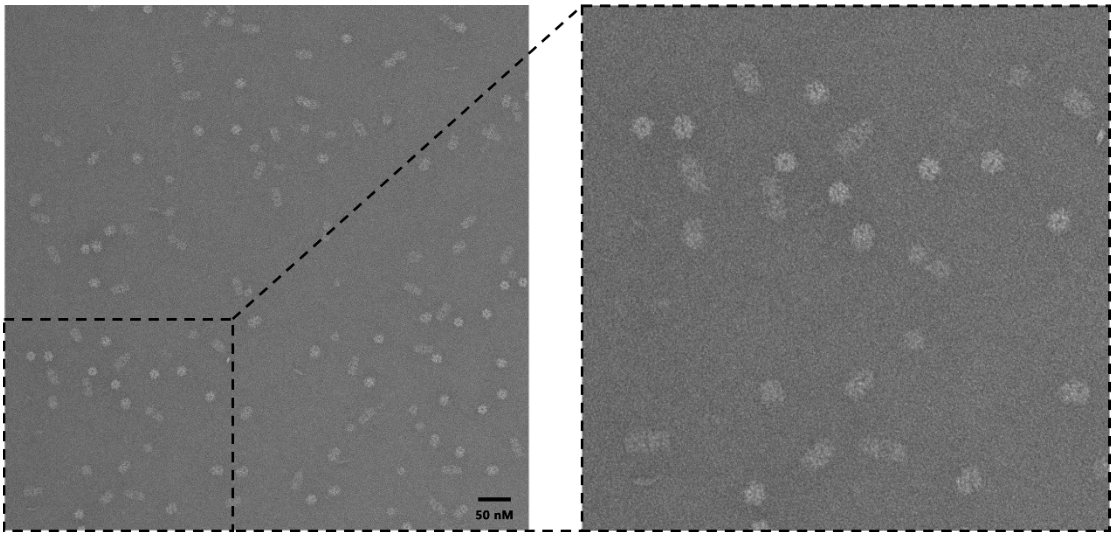

Fig. S4: A typical negative staining CCD image of the full-length apo-form C962R protein.

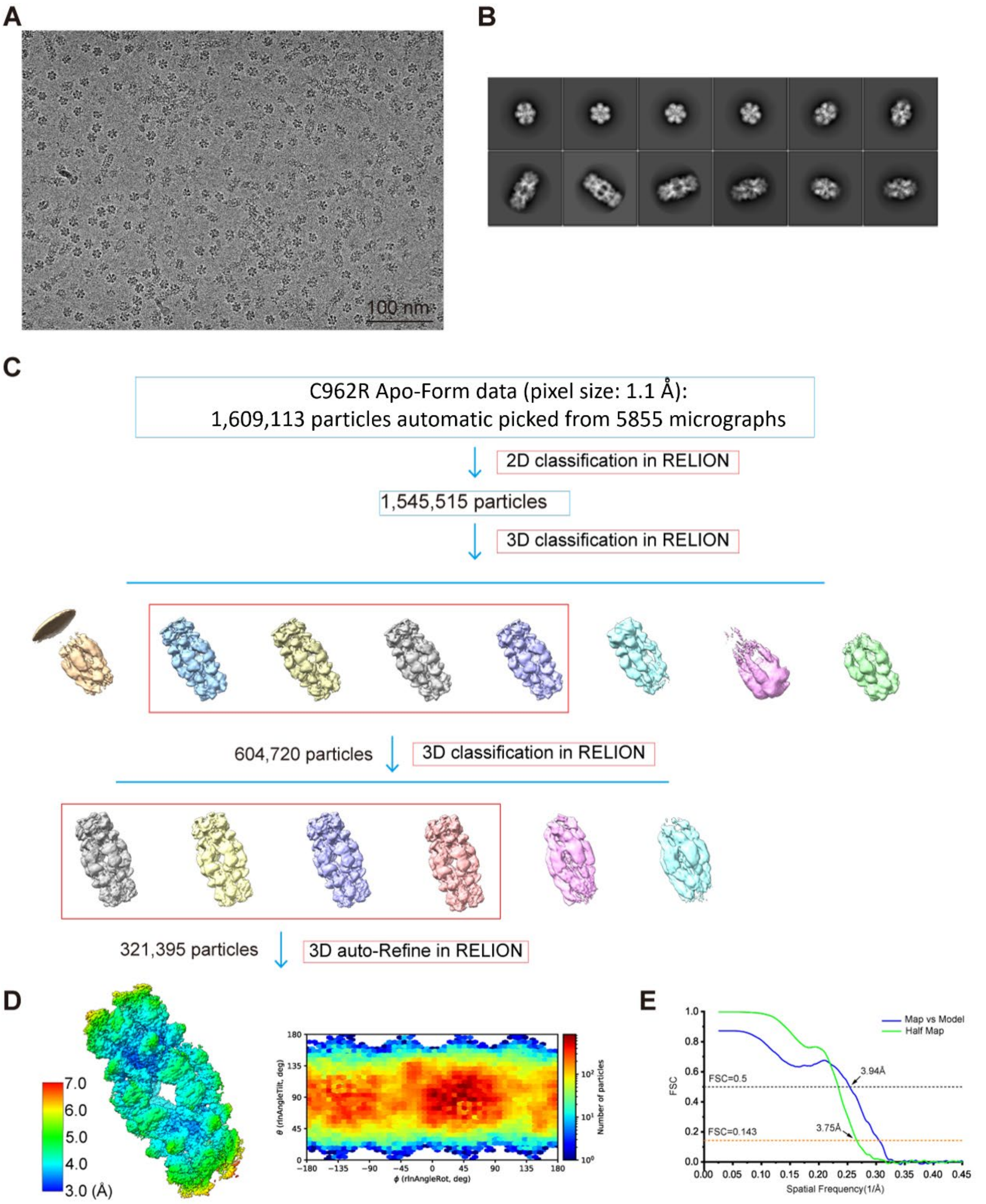

Fig. S5: Cryo-EM data processing of the apo-form C962R structure. (**A**) A representative cryo-EM image of the apo C962R structure. (**B**) Representative 2D-classification images of the apo C962R structure. (**C**) Workflow of cryo-EM data processing. (**D**) Particle distribution and final electron density map colored according to the local resolution of the apo C962R structure. (**E**) Gold-standard Fourier shell correlation (GSFSC) of the final map of the apo C962R structure.

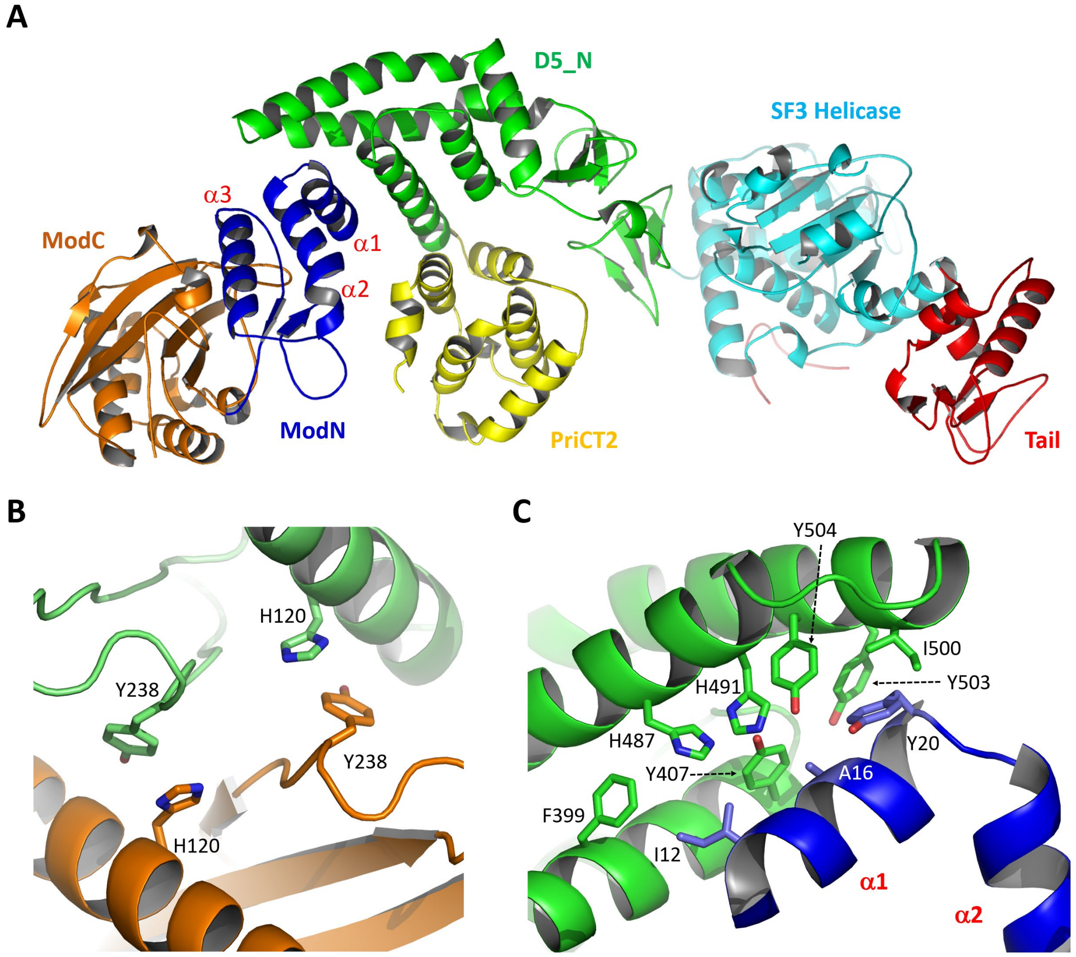

Fig. S6: C962R monomers in the apo-form structure. (**A**) Overall folding of single C962R monomer. The AEP, PriCT2, D5_N, SF3 helicase and Tail domains are colored in blue-and-brown, yellow, green, cyan and red, respectively. (**B**) Conformations of the residues involved in the dodecamerization of C962R. (**C**) The hydrophobic residues located at the interfaces of the AEP and D5_N domains.

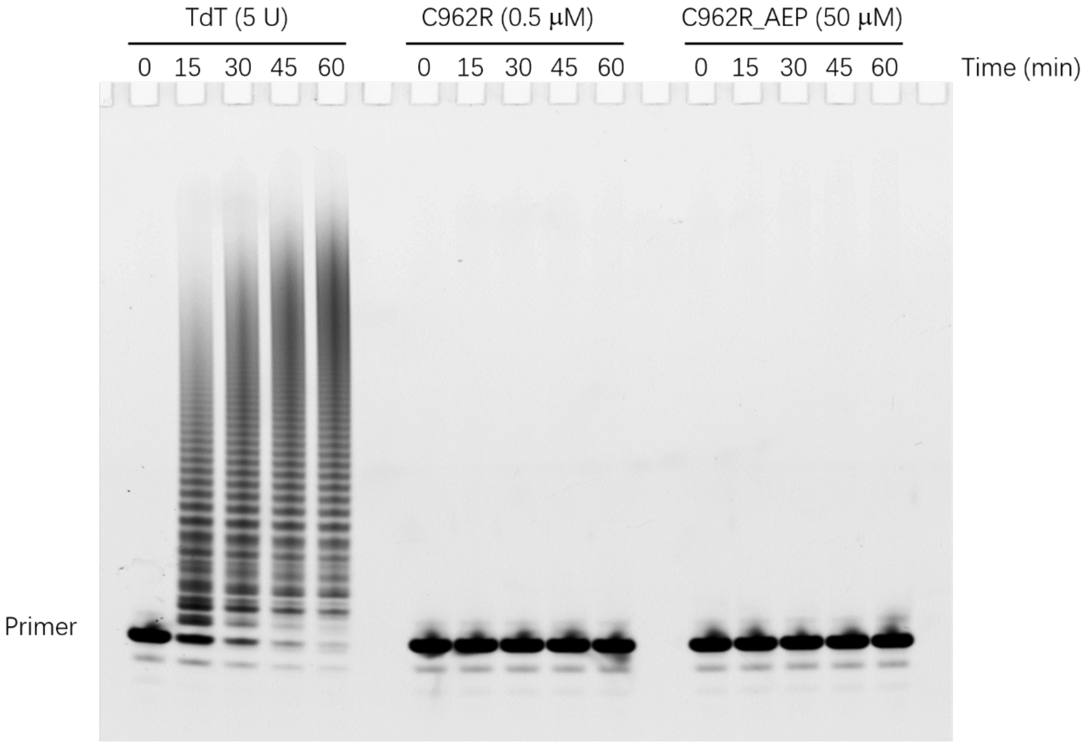

Fig. S7: Investigation of the terminal deoxynucleotidyl transferase activity of the full-length protein and the isolated AEP domain of C962R. The calf thymus Tdt protein is utilized as positive control.

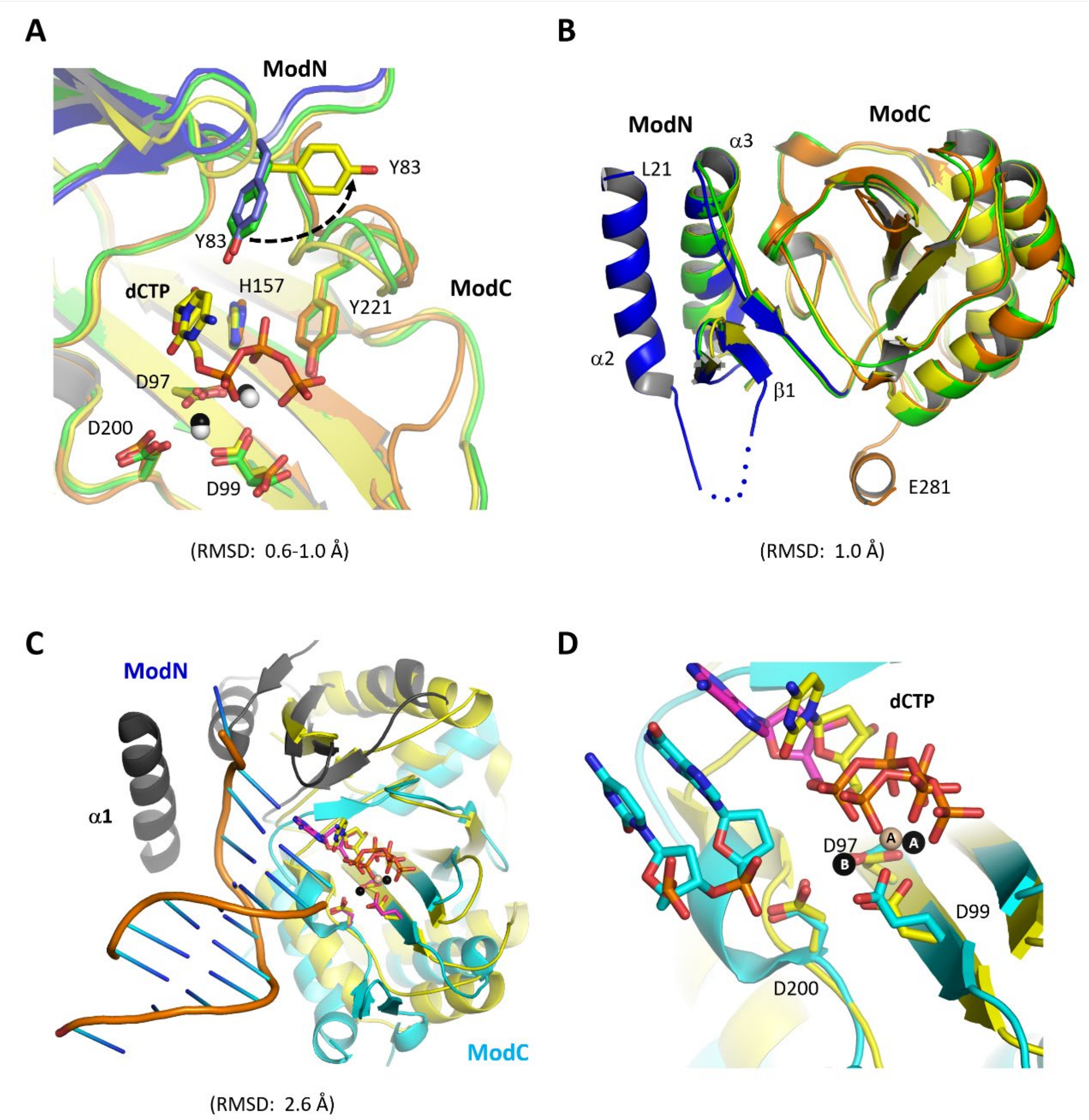

Fig. S8: Comparison of the AEP domain structures. (**A**) Superposition showing the conformational changes of Tyr83 and Asp99 in the apo-form AEP, AEP-Mn^{2+} and AEP-dCTP-Mn^{2+} structures of C962R. (**B**) Superposition showing the disordering of the α2 helices in the AEP-Mn^{2+} and AEP-dCTP-Mn^{2+} structures of C962R. (**C-D**) Comparison of the AEP domain structures of C962R and *Hs*PrimPol. The C-atoms are colored in green and yellow in the AEP-Mn^{2+} and AEP-dCTP-Mn^{2+} structures of C962R, respectively. The ModN and ModC subdomains are color in blue and brown in the apo-form AEP structure. For the *Hs*PrimPol structure, the ModN subdomain is colored in black. The C-atoms of the ModC subdomain, the primer and the incoming dATP are colored in cyan, cyan and magenta, respectively.

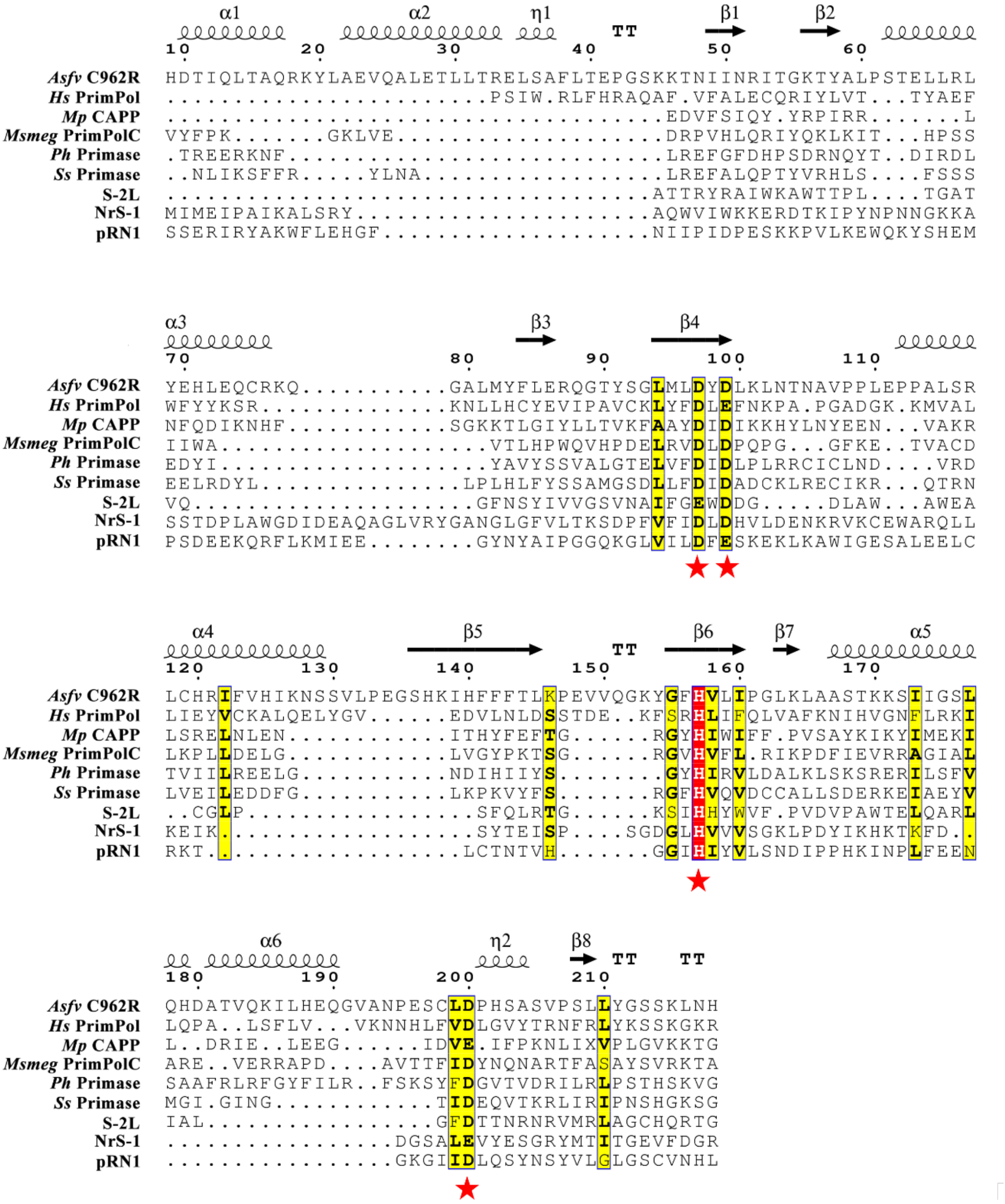

Fig. S9: Structure-based multiple sequence alignment of the AEP domain of C962R and the homologous proteins. The amino acid sequences of C962R AEP domain, eight proteins from the *Hs*PrimPol (PDB_ID:5L2X), *Mp*CAPP (PDB_ID: 7NQF), *Msmeg*PrimPolC (PDB_ID: 6SA0), *Ph*Primase (PDB_ID: 1V33), *Ss*Primase (PDB_ID:1ZT2), *Cyanophage* S-2L PrimPol (PDB_ID: 6ZP9), *Deep sea vent phage* NrS-1 polymerase (PDB_ID: 6JON) and *Sulfolobus islandicus* pRN1 (PDB_ID: 1RO0) were aligned. The secondary structure elements of C962R AEP domain are listed on the top of the alignment. The catalytic residues are indicated by the red asterisks.

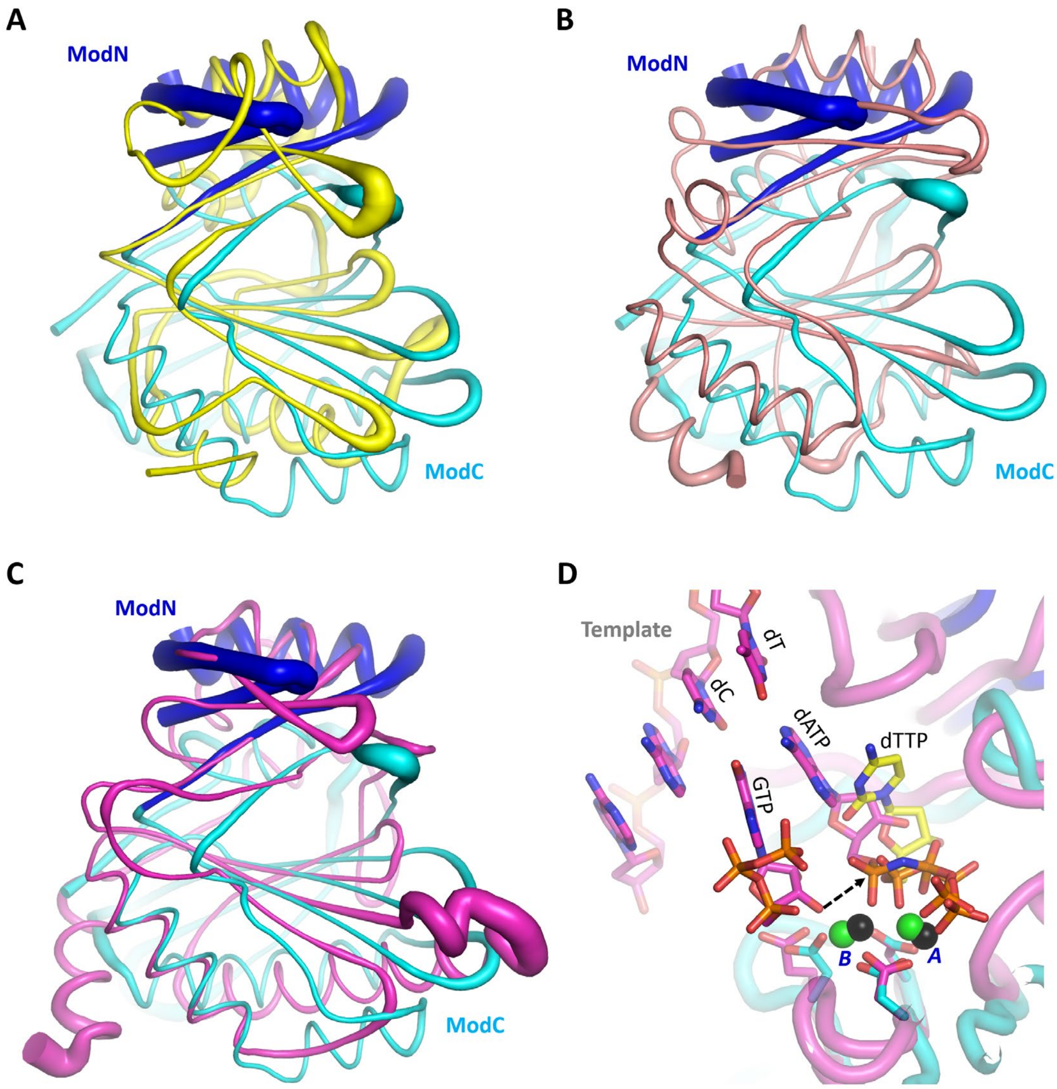

Fig. S10: Comparison of the AEP domain structures of C962R and the homologous proteins. (**A**) Superposition of the AEP domains of C962R and NrS-1 polymerase (PDB_ID: 6JON). (**B**) Superposition of the AEP domains of C962R and S-2L PrimPol (PDB_ID: 6ZP9). (**C-D**) Comparison of folding, Cation and dNTP binding by the AEP domains of C962R and the CAPP protein (PDB_ID: 7QAZ). The ModN and ModC of C962R AEP domains are colored blue and cyan, respectively. The cations and the C-atoms of dCTP bound by the AEP domain of C962R are colored in black and yellow, respectively.

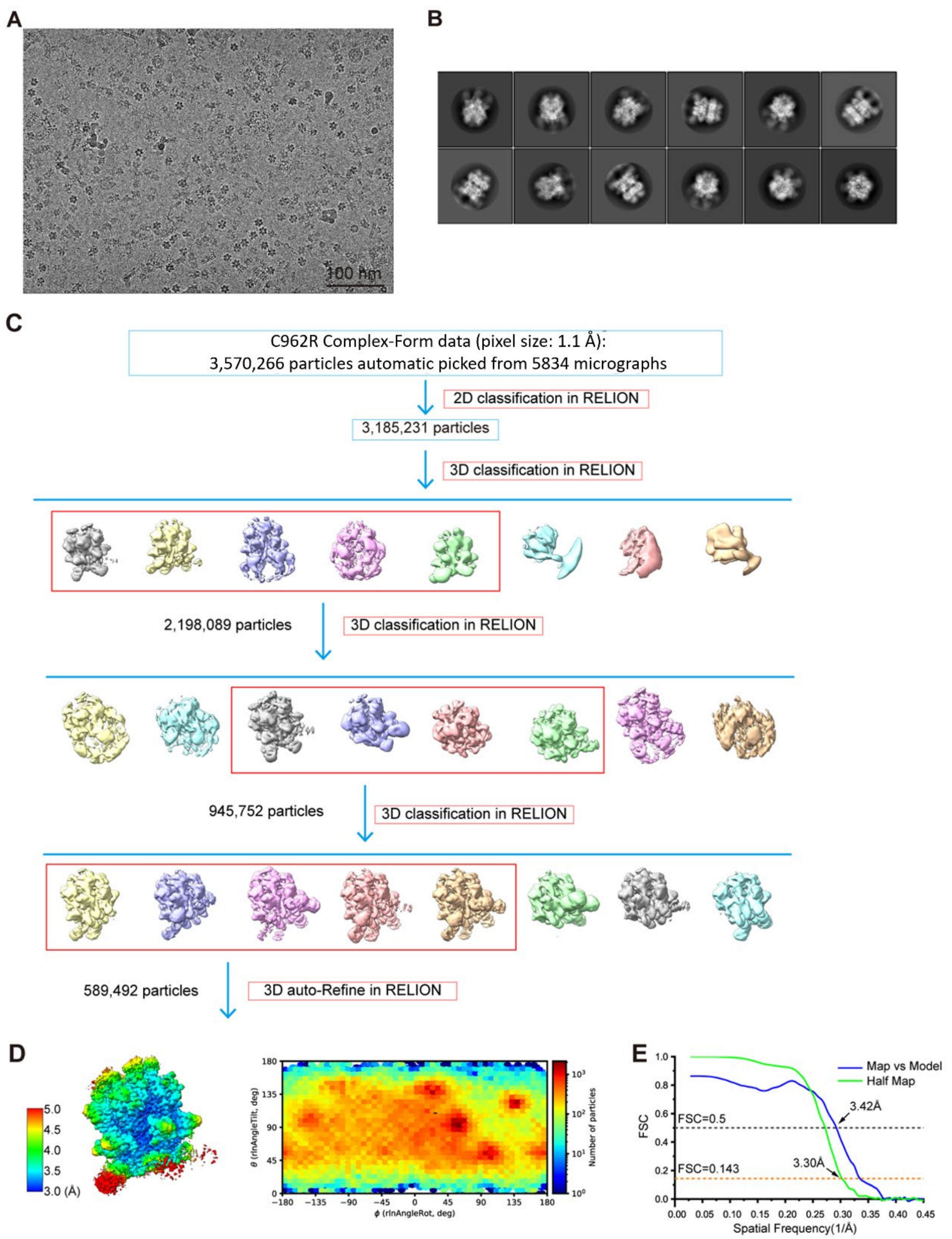

Fig. S11: Cryo-EM data processing of the C962R-DNA-AMPPNP complex structure. (**A**) A representative cryo-EM image of the complex structure. (**B**) Representative 2D-classification images of the complex structure. (**C**) Workflow of cryo-EM data processing. (**D**) Particle distribution and final electron density map colored according to the local resolution of the complex structure. (**E**) Gold-standard Fourier shell correlation (GSFSC) of the final map of the complex structure.

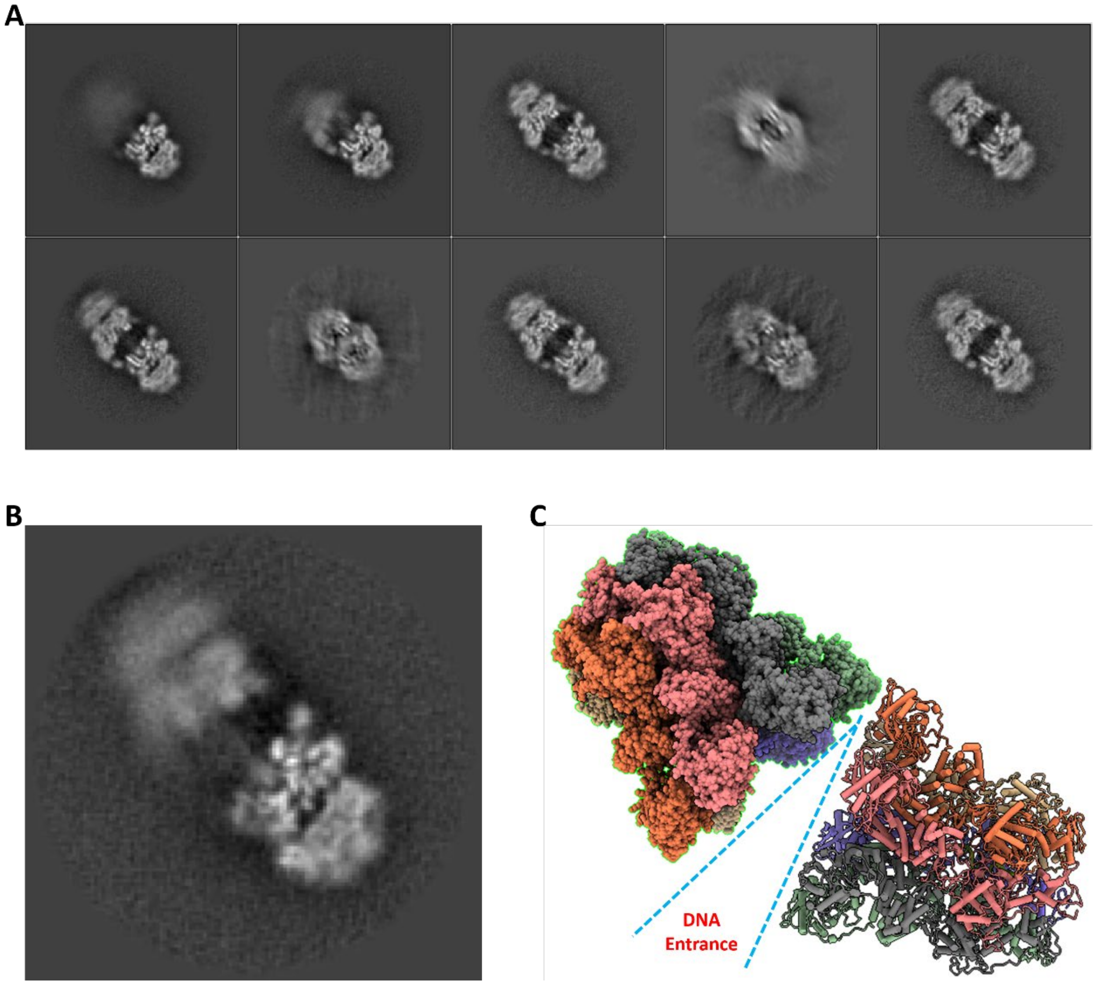

Fig. S12: Electron microscopy analysis of the C962R-DNA-AMPPNP complex structure. (**A**) The three-dimensional class averages of C962R. (**B-C**) Enlarged image and model for two tilted C962R hexamers in the complex structure.

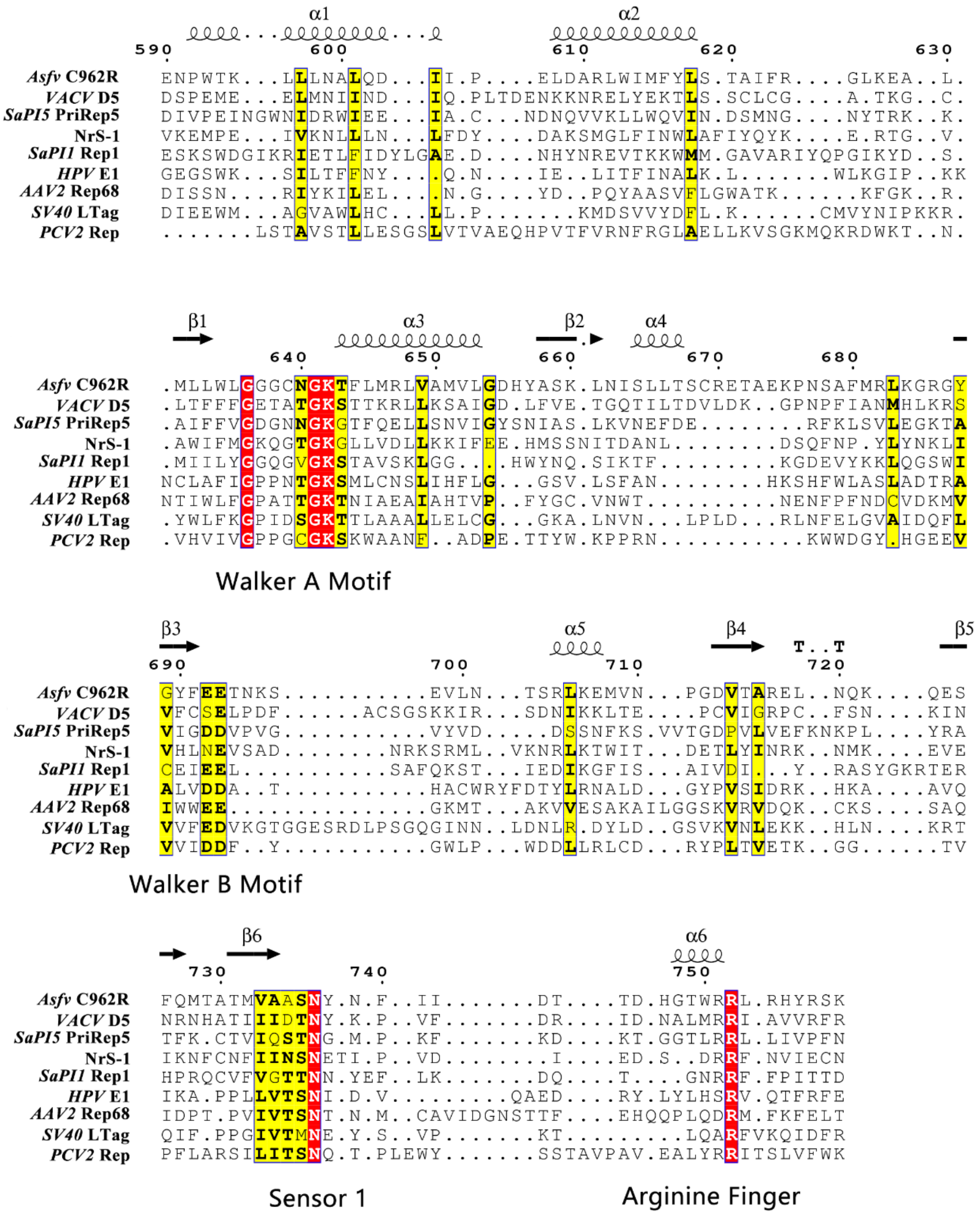

Fig. S13: Structure-based multiple sequence alignment of the SF3 helicase domain of C962R and the homologous proteins. The amino acid sequences of C962R SF3 helicase domain, eight proteins from the VACV D5 (PDB_ID: 8APL), SaPI5 PriRep5 (PDB_ID: 7OLA), NrS-1 polymerase (PDB_ID: 6K9C), SaPI Rep1 (7PDS), HPV E1 (7APD), AAV Rep68 (PDB_ID: 7JSF), SV40 LTag (PDB_ID: 4GDF) and PCV2 Rep (PDB_ID: 7LAR) were aligned. The secondary structure elements of C962R SF3 helicase domain is listed on the top of the alignment.

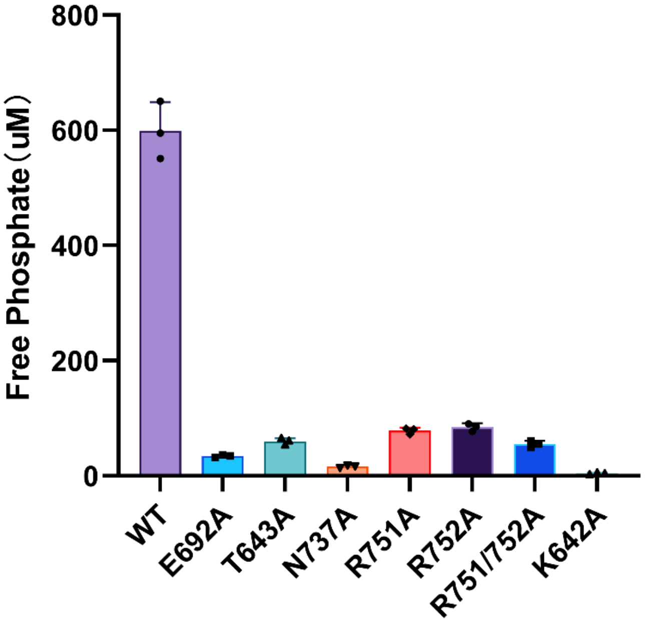

Fig. S14: Comparison of the ATPase activities of WT and mutated C962R proteins.

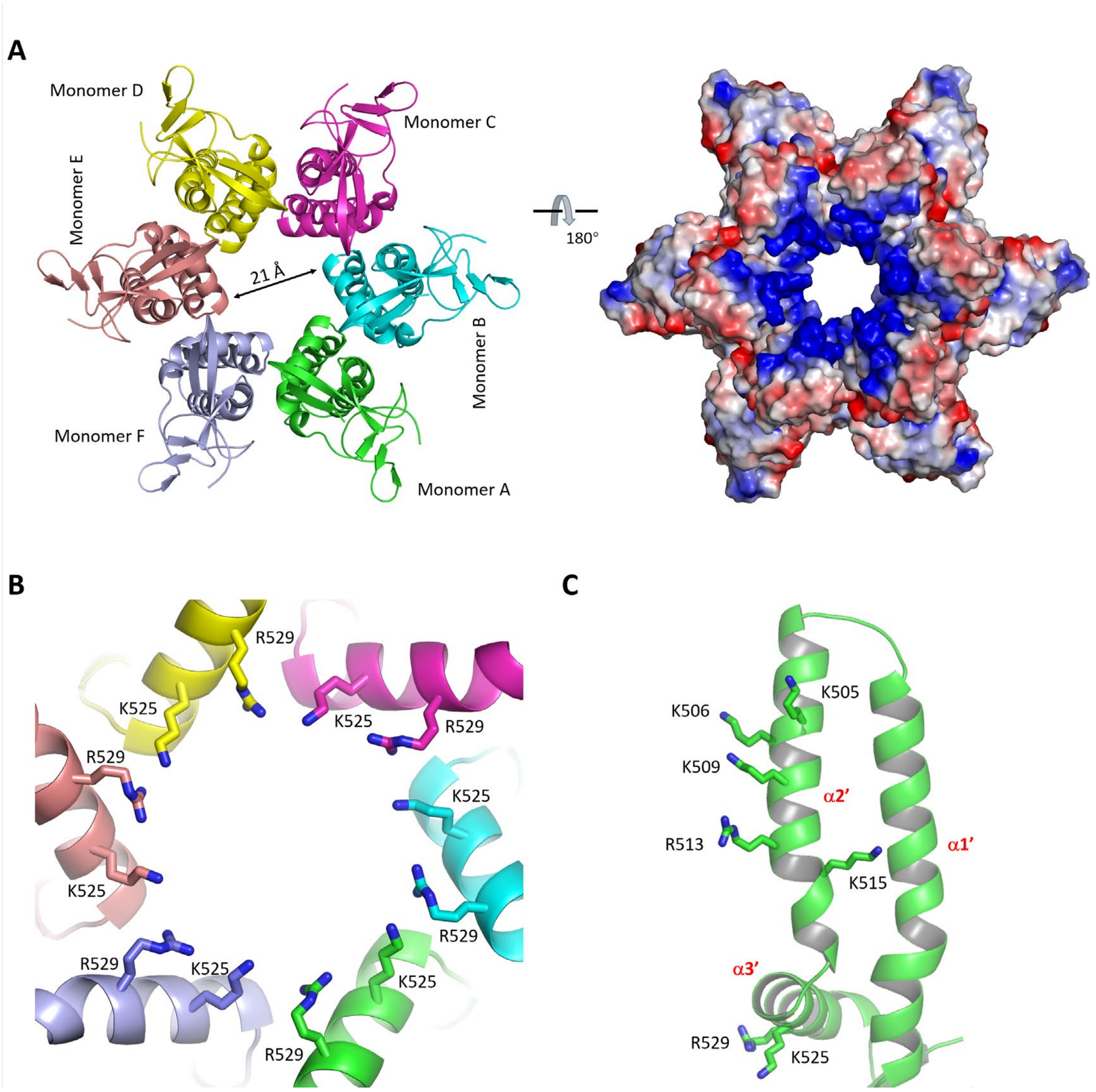

Fig. S15: Structural analysis of the D5_N domain of C962R. (**A**) Cartoon and surface presentations of the D5_N domains. (**B**) The detailed conformations of Lys525 and Arg529 of the D5_N domains. (**C**) The detailed conformations of Lys505, Lys506, Lys509, Arg513 and Lys517 located on the second helix of the D5_N domain.

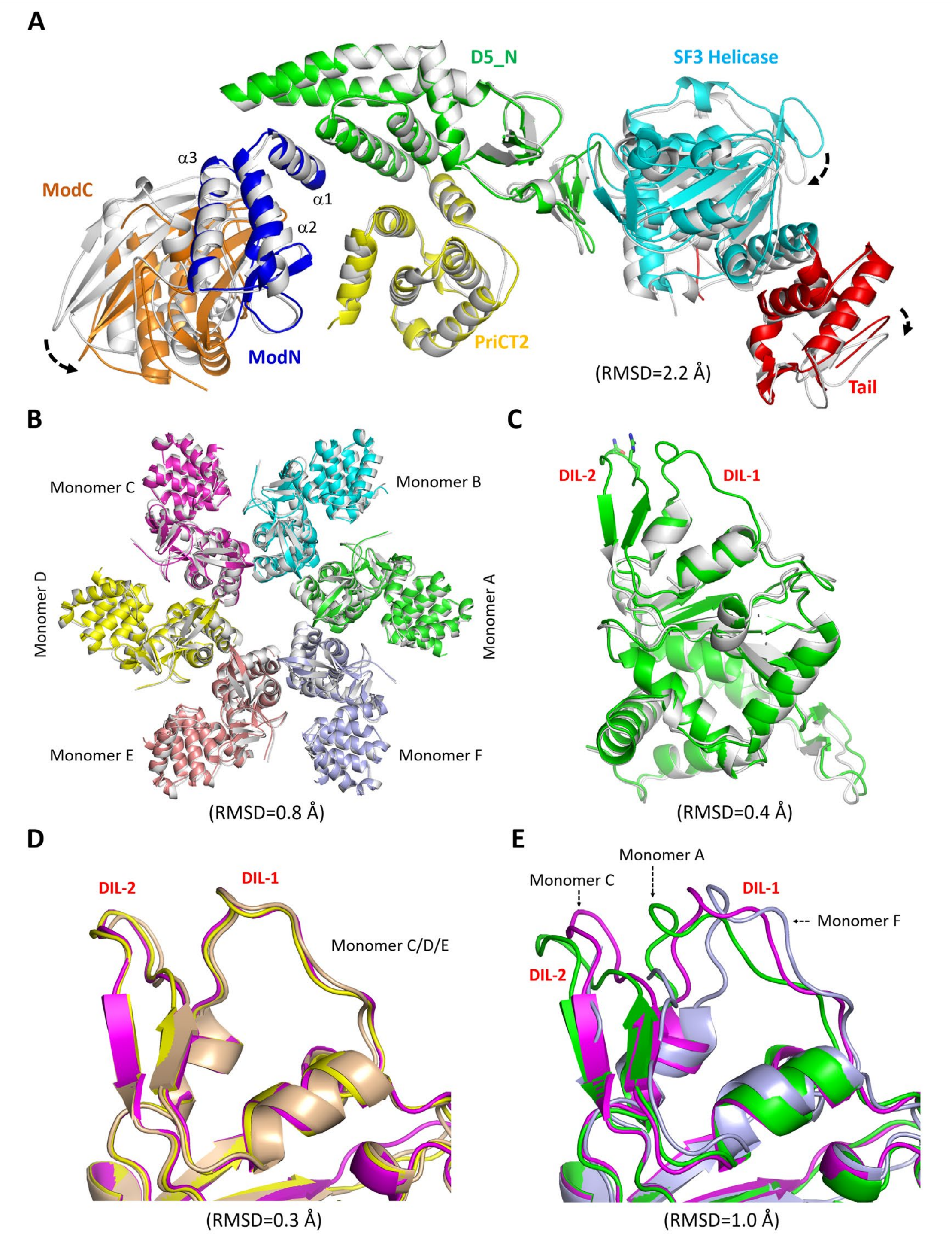

Fig. S16: Comparison between the apo and complexed structures of C962R. (**A**) Superposition of the full-length protein. (**B**) Superposition of the Pri_CT2/D5_N domains. (**C-E**) Superposition of the Helicase domains. All C962R monomers are colored in white in the apo-form structure. In the complex structure, the monomers A-F are colored in green, cyan, magenta, yellow, pink and light blue, respectively.

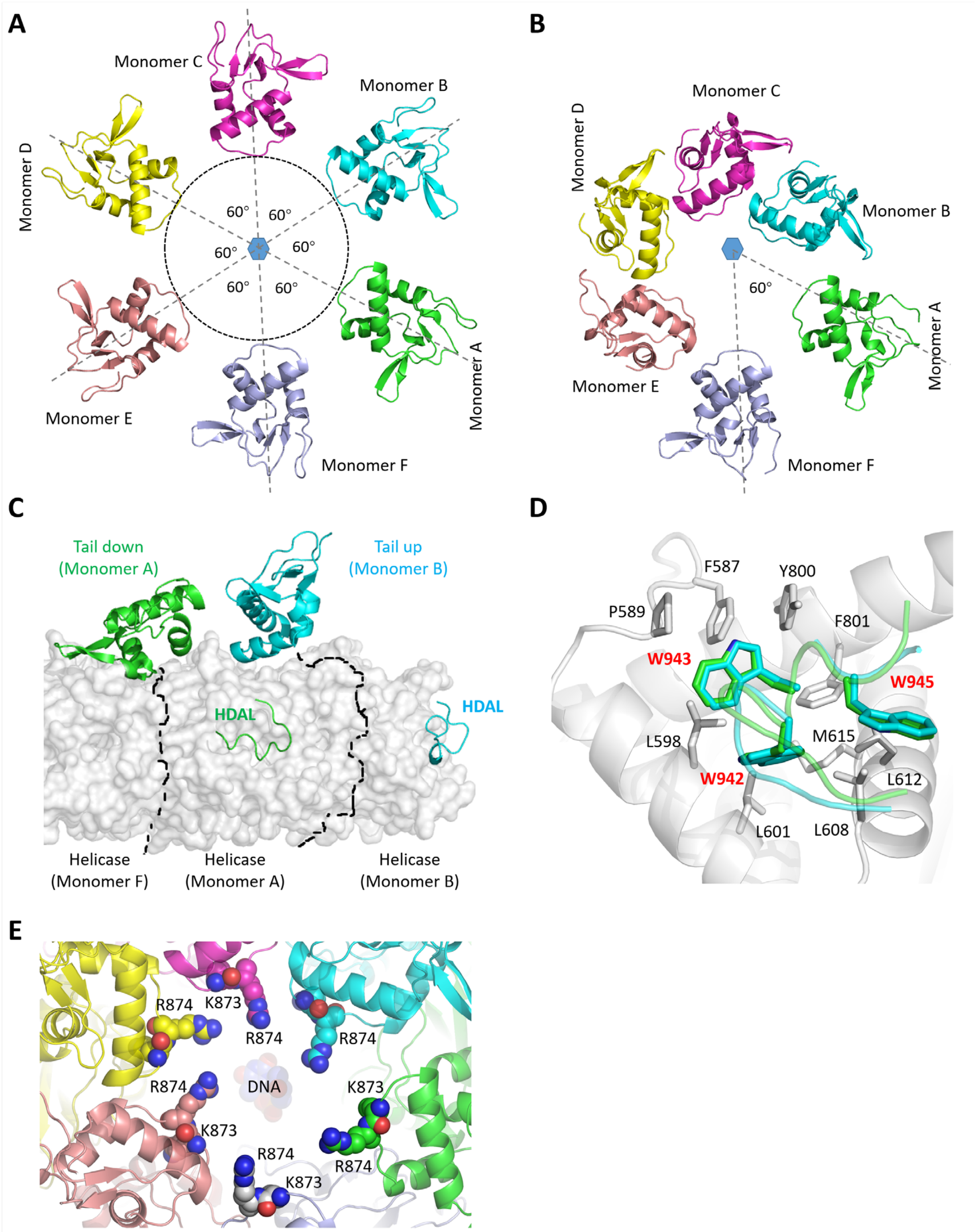

Fig. S17: Structural analysis of the Tail domain of C962R. (**A-B**) Arrangement of the Tail domains in the apo- and the complexed structures, respectively. (**C**) Conformations of the Tail domains and the HDAL loops in the complex structure. (**D**) Superposition showing the conserved interactions between the HDAL loop and the Helicase domain. (**E**) The detailed conformations of Lys873 and Arg874 in the complex structure.

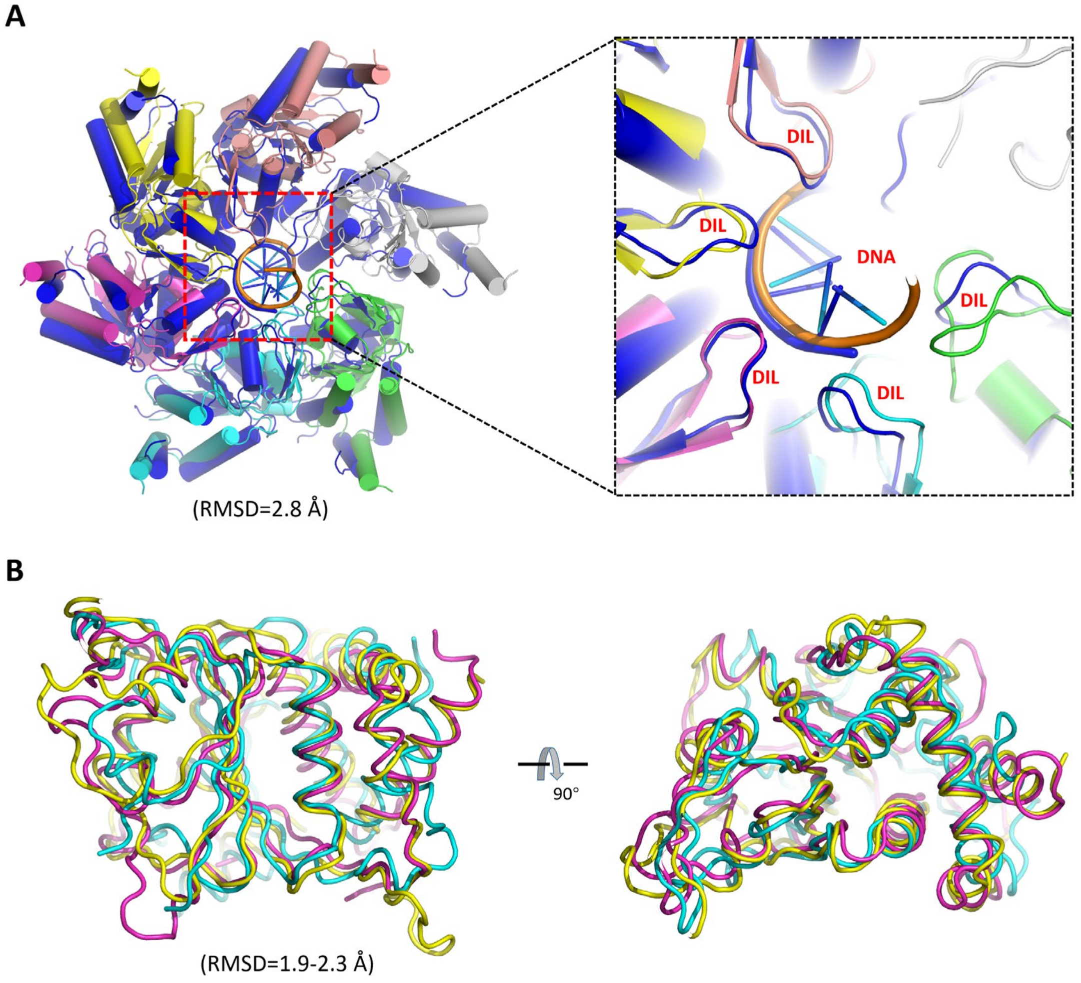

Fig. S18: Comparison of the helicase domains of C962R and the homologous proteins. (**A**) Superposition of the DNA-bound C962R and HPV E1 protein (PDB_ID: 2GXA). The DNA and all E1 protomers are colored in blue in the E1-DNA complex. The DNA and C962R protomers are colored differently in the C962R-DNA complex. (**B**) Superposition of the helicase domains of C962R, NrS-1 polymerase (PDB_ID: 6LRB) and VACV D5 (PDB_ID: 8APL), which are colored in yellow, cyan and magenta, respectively.

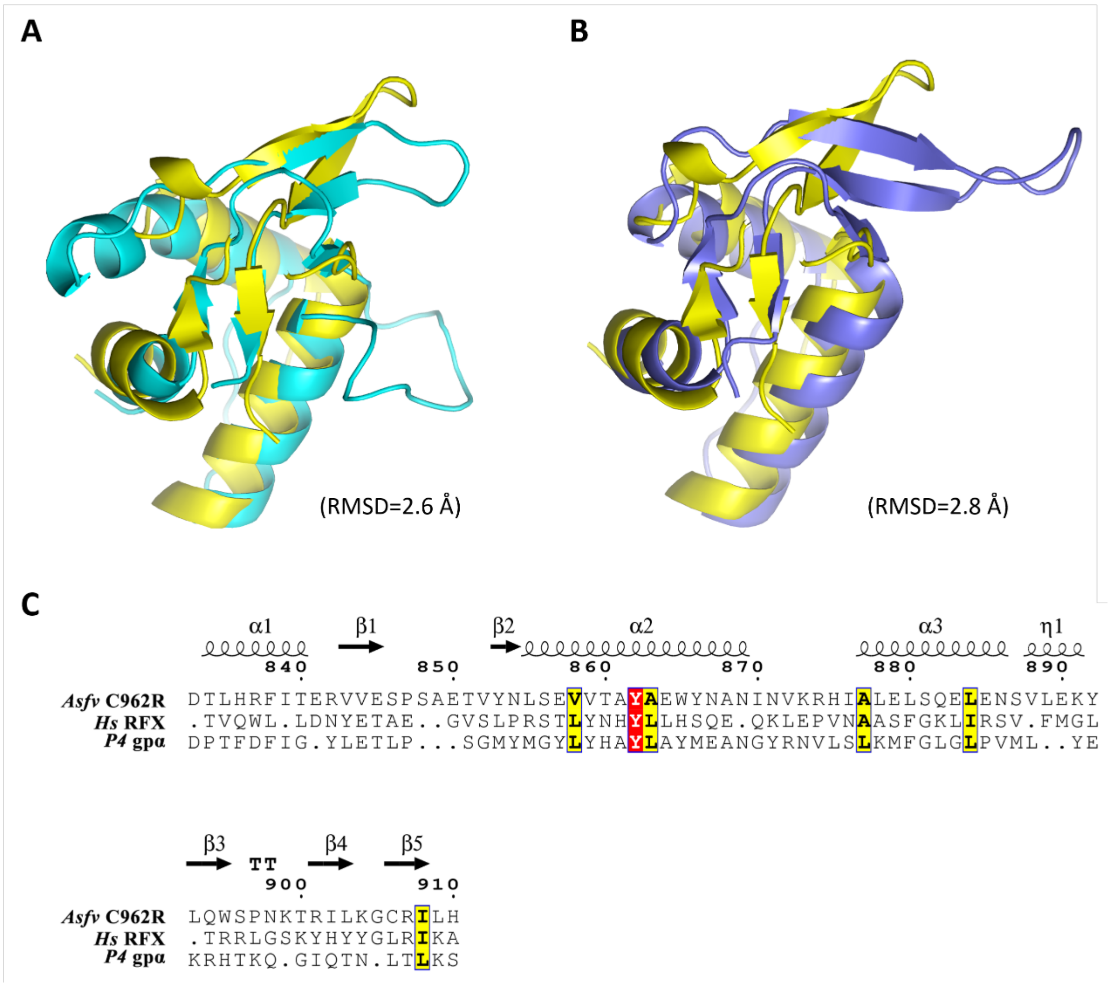

Fig. S19: Comparison of the Tail domain of C962R and other DNA interacting domains. (**A**) Superposition of the Tail domain of C962R and the OBD domain of P4 gpalpha protein (PDB_ID: 1KA8). (**B**) Superposition of the Tail domain of C962R and the DBD domain of Rfx1 (PDB_ID: 1DP7). (**C**) Structure-based multiple sequence alignment of C962R Tail domain, P4 gpalpha OBD domain and Rfx1 DBD domain.

Table S1: Coden-optimized cDNA sequence of C962R

The optimized cDNA sequence of C962R (from 5’ to 3’) [a]

GGATCCATGCGCGAGGAAAGCTGGGAGGAACACGATACCATTCAGCTGACCGCCCAGCGCAAATATCTCGCGGAGGTGCAA
GCGCTCGAAACGCTGCTGGCGCGTGAACTGAGCGTTTTTCTCACCGAACCGGGCAGCAAGAAGACGAACATCATCAACCGC
ATCACGGGTAAGACCTATGCGCTCCCAAGCACCGAGCTGCTCCGCTTCTATGAGCATCTGGAACAGTGCCGCAAGCAAGGC
GCGCTGATGTACTTTCTGGAGCGCCAAGGCACGTATAGCGGTCTCATGCTGGATTACGATCTGAAGCTCAACACGAACGCG
GCGCCAAGTCTGGAGAGTAGTGTTCTGAGTCGCCTCTGCCACCGTATCTTCGTGCACATCAAGAACAGCAGCGTGCTCCCG
GAAGGCAGCCACAAAATTCACTTTTTCTTTACGCTCAAGCCGGAGGCGGTTCAAGGCAAGTACGGCTTCCACGTTCTGATT
CCGGGTCTGAAGATGGCCGCCAGCACCAAGAAGAGCATTATCGCCAGTCTCCAGCACGACGCGACCGTGCAGAAAATCCTC
CATGAACAAGGCGTGGCGAATCCGGAAAGTTGTCTGGACCCACACAGCGCCAGCGTTCCGAGTCTGCTGTATGGTAGTAGC
AAGCTGAACCATCGCCCATACCAGCTCAAAACGGGTTTCGAGCTCGTGTTCGATAGCAGCGATCCGGACTATATCCCGATC
CATCAGATCAAAAATATCGAGAGCTACAATCTCGTGAGCGAACTGAGCCTCACCAATGAGCAAGGTAGTCTGGTTCGCCCA
GTGTACTGTGCCGCGGACATCGCGGCCGAAAAAGAGGAAGAAATCCCGGCCGATGATCACAGTCTGAGCATTCTGATGCTC
CACGATCCGGAAGCCCGCTATCTGCACAAAATCCTCAATCTGCTGCCACCGGAATACTATGTTGAGTACCCGCTGTGGAGC
AACGTGGTGTTCGCCCTCGCGAATACGAGCGCCAACTACCGTCCGCTCGCGGAATGGTTCAGTCAGAAGTGCCCGGAGAAA
TGGAACACCGGCGGCAAAGAGAAGCTGGAAAAGCTGTGGAATGACGCGAGCCGCCATACCGAGAAGAAAATCACGAAGCGC
AGCATTATGTATTGGGCGCACAAACACGCCCCACAGCAGTACAAGGAGATCGTTGAACAAGGCTATTTCAGCATTCTGGCG
GAATATGTGTATAGTTACAATGGTACGCTGGAACACTACATGATTGCCAAAGTGATCTACGCCATGATGGGCAATAAATTC
GTGGTGGATGTGGACAGCAACGGCAAGTACGTGTGGTTTGAATTCGTGCTGCCGGGTCAACCGATGAATCAAGGCGAGATC
TGGAAGTGGCGCAAAGAAGTTAATCCGGACGAACTGCACATTTACATCAGCGAAAATTTCAGTCGCGTGATGGACCGCATC
ACCGAGCACATCAAGTACCATCTGAGCCAGCCGCACGAAACCAACATTCTCAACTACTATAAGAAGCTGCTGAAAGCCTTT
GAGCGCAGCAAGAGCAAGATTTTTAATGATAGCTTCAAGAAAGGCGTGATTCGCCAAGCCGAATTTCTCTTCCGCCAACGC
AGCTTCATTCAGACGCTGGATACCAATCCGTATCTGCTCGGTGTTGGCAACGGCGTTCTGAGCATTGAAACCATCCCGGCC
AAGCTGATCAATCACTTCCACGAGCACCCGATTCACCAGTACACGCACATCTGCTACGAGCCATTTAATCCGGAGAATCCG
TGGACGAAGCTGCTGCTGAATGCGCTGCAAGATATCATCCCAGAGCTCGACGCGCGCCTCTGGATCATGTTTTACCTCAGC
ACGGCGATCTTCCGCGGTCTGAAAGAAGCCCTCATGCTGCTGTGGCTCGGCGGTGGCTGCAACGGTAAGACGTTCCTCATG
CGTCTGGTTGCCATGGTGCTGGGCGACCATTACGCCAGCAAGCTCAACATTAGTCTGCTCACGAGTTACCGCGAAACGGCC
GAGAAGCCAAATAGCGCCTTCATGCGTCTGAAGGGTCGTGGTTACGGCTATTTCGAGGAGACGAACAAAAGCGAGATCCTC
AATACCAGCCGTCTGAAAGAGATGGTGAACCCGGGTGATGTGACGGCCCGTGAACTGAACCAGAAGCAAGAAAGCTTTCAG
ATGACCGCCACCATGGTGGCGGCGAGCAACTACAATTTCATCATCGACACCACGGACCACGGCACGTGGCGTCGTCTCCGC
CACTATCGCAGCAAGGTTAAATTCTGCCATAACCCGGACCCGAACAACAGTTACGAGAAGAAAGAAGATCCGCGTTTCATC
CACGAGTACATTATGGACCCGAACTGCCAGAATGCGTTCTTCAGTATCCTCGTGTATTTTTGGGAGAAGCTCCAGAAAGAG
TACAATGGCCAGATCAAGAAGGTTTTCTGCCCGACCATCGAGAGTGAGACGGAGGCCTACCGTAAGAGTCAAGATACGCTG
CATCGCTTTATCACCGAACGTGTGGTTGAGAGTCCGAGCGCGGAAACCGTGTACAATCTGAGCGAGGTTGTTACCGCGTAC
GCCGAATGGTACAACGCCAATATCAACGTGAAACGCCACATCGCGCTGGAACTCAGTCAAGAACTGGAGAACAGTGTGCTG
GAAAAATACCTCCAGTGGAGCCCGAACAAGACCCGCATTCTGAAGGGTTGCCGCATTCTCCACAAGTTTGAGACGCTGCAA
CCGGGCGAAAGTTACATTGGCGTGAGTAGCACCGGTACGCTGCTGAACACGCCGATCTGCGAGCCAAAGAATAAGTGGTGG
GAGTGGAGCCCAAATCCGAGCGCGCCACCGGAGAAAGAAGCGAGTGCGCCGACCCCATAACTCGAG

[a]: ***GGATCC*** and ***CTCGAG*** at the 5'-end and 3'-end are Bam HI and Xho I recognition sequence.

Table S2: Primers used for mutant or truncated C962R construction.

Name	Sequence (5'-3')
WT-F	AGAGAACAGATTGGTGGATCCATGCGCGAGGAAAGCTGG
WT-R	GTGGTGGTGGTGGTGCTCGAGTTATGGGGTCGGCGCACT
21-F	AGAGAACAGATTGGTGGATCCCTCGCGGAGGTGCAAGCG
272-R	GTGGTGGTGGTGGTGCTCGAGTTAGGCACAGTACACTGGGCG
285-R	GTGGTGGTGGTGGTGCTCGAGTTAGGCCGGGATTTCTTCCT
K642A-F	AACGGTGCCACGTTCCTCATGCGTCTGGTTGC
K642A-R	AGGAACGTGGCACCGTTGCAGCCACCGCCGAG
K439A-F	GCCTACGTGTGGTTTGAATTCGTGCTGCCGGG
K439A-R	TCAAACCACACGTAGGCGCCGTTGCTGTCCACATCC
K525A-F	TGATAGCTTCAAGGCCGGCGTGATTCGCCAAGCC
K525A-R	CGGCCTTGAAGCTATCATTAAAAATCTTGCTC
R529A-F	TGATTGCCCAAGCCGAATTTCTCTTCCGCCAA
R529A-R	TTCGGCTTGGGCAATCACGCCTTTCTTGAAGCTATC
T643A-F	GGTAAGGCCTTCCTCATGCGTCTGGTTGCCAT
T643A-R	ATGAGGAAGGCCTTACCGTTGCAGCCACCGCC
K675A-F	GCCCCAAATAGCGCCTTCATGCGTCTGAAGGG
K675A-R	AAGGCGCTATTTGGGGCCTCGGCCGTTTCGCGGTA
E692A-F	CTATTTCGCCGAGACGAACAAAAGCGAGATCC
E692A-R	TCGTCTCGGCGAAATAGCCGTAACCACGACCC
R717A-F	GCCGAACTGAACCAGAAGCAAGAAAGCTTTCA
R717A-R	TTCTGGTTCAGTTCGGCGGCCGTCACATCACCCGG
N720A-F	AACTGGCCCAGAAGCAAGAAAGCTTTCAGATG
N720A-R	TTGCTTCTGGGCCAGTTCACGGGCCGTCACAT
N737A-F	GCGAGCGCGTACAATTTCATCATCGACACCACG
N737A-R	AAATTGTACGCGCTCGCCGCCACCATGGTGGCGGTCATCTGAA AGCTTT
R751A-F	GCGCGTCTCCGCCACTATCGCAGCAAGGTTAA
R751A-R	TAGTGGCGGAGACGCGCCCACGTGCCGTGGTCCGT
R752A-F	GTGCGCTCCGCCACTATCGCAGCAAGGTTAAA
R752A-R	ATAGTGGCGGAGCGCACGCCACGTGCCGTGGTC
R751A/R752A-F	GCGGCGCTCCGCCACTATCGCAGCAAGGTTAAA
R751A/R752A-R	ATAGTGGCGGAGCGCCGCCCACGTGCCGTGGTCCGT
K873A/R874A-F	AATATCAACGTGGCCGCCCACATCGCGCTGGAACTCA
K873A/R874A-R	GGCGGCCACGTTGATATTGGCGTTGTACCATTC
K/R5A-F	CTTTGAGGCCAGCAAGAGCGCCATTTTTAATGATAGCTTCAAG AAAGGC
K/R5A-R	TCTTGCTGGCCTCAAAGGCGGCCAGCAGGGCGGCATAGTAGTT GAGAATGTTGGTTTCG

Table S3: Cryo-EM data collection, processing, model refinement and validation statistics

**Structure**	Apo-Form	Complex-Form
PDB ID	8IQH	8IQI
EMDB ID	EMD-35670	EMD-35671
**Data collection and Processing**		
Microscope	Titan Krios	
Detector	Gatan K3	
CS (mm)	0.01	0.01
Magnification	64K	64K
Pixel size (Å)	1.10	1.10
Electron dose ($e^-/Å^2$)	50 (32 frames)	50 (32 frames)
Defocus range (μm)	-1.5-2.0	-1.5-2.0
Micrograph Number	5934	6688
**Reconstruction**		
Software	RELION-3.1.0	
Particles picked	1609113	3570266
Particles refinement	321395	589492
Symmetry	C1	C1
Resolution (Å)	3.67	3.32
Sharpening B-factor ($Å^2$)	-99.07	-45.00
**Refinement**		
Software	Phenix-1.19-4092	
Model Composition		
Number of atoms	77480	40438
Protein residues	10535	5488
Nucleotides	0	10
Ligand (Mg^{2+}/AMPPNP)	0/0	4/6
B factors (Protein/DNA/AMPPNP)	117.34/0/0	131.68/132.19/114.51
Bonds RMSD		
Bonds lengths (Å)	0.007	0.006
Bonds angels (°)	0.771	1.205
**Validation**		
MolProbity score	1.22	1.20
Clash score	3.14	2.88
Rotamer outliers (%)	0.45	0.64
C-beta outliers (%)	0.01	0.00
Ramachandran plot		
Favored (%)	97.39	97.39
Allowed (%)	2.60	2.61
Outlier (%)	0.01	0.00
Model vs. Data		
CC mask/box	0.79/0.86	0.82/0.87

Table S4: Crystal data collection and refinement statistics.

Structure	Apo-form AEP	AEP-Mn^{2+}	AEP-dCTP -Mn^{2+}
PDB ID	8IQB	8IQC	8IQD
**Data collection** [a]			
Space group	$P2_1$	C2	C2
Cell parameter:			
a, *b*, *c* (Å)	43.8, 73.6, 78.9	128.4, 43.9, 87.6	209.2, 42.8, 128.2
α, β, γ (°)	90.0, 95.4, 90.0,	90.0, 93.3, 90.0	90.0, 125.0, 90.0
Wavelength (Å)	1.0000	1.0000	1.0000
Resolution (Å)	53.72-2.58	30.00-1.80	100.31-2.39
High-resolution shell (Å)	2.72-2.58	1.86-1.80	2.52-2.39
Completeness (%)	98.5 (99.5)	98.5 (90.9)	97.6 (97.4)
Redundancy	3.0 (2.9)	5.0 (3.0)	3.2 (3.3)
R_{merge} (%)	11.6 (57.7)	9.6 (45.5)	6.5 (45.4)
I/σ(I)	5.2 (2.4)	15.1 (1.73)	8.9 (2.4)
**Refinement**			
Resolution (Å)	20.71-2.58	29.55-2.00	85.62-2.39
No. of reflections	28321	32825	36576
R_{work} (%) / R_{free} (%)	21.7/26.4	24.4/27.7	24.1/28.3
No. of atoms			
Protein	3677	3695	6931
Water	13	163	24
Mn^{2+}	0	4	11
dCTP	0	0	78
R.m.s. deviations			
Bond length (Å)	0.003	0.002	0.003
Bond angle (°)	0.647	0.500	0.601
Ramachandran plot (%)			
Most favorable	97.97	97.59	98.91
Additional allowed	2.03	2.41	0.98

[a]: Values in parentheses are for the high-resolution shell.

Table S5: DNAs used in *in vitro* assays.

Name	Sequence（5'-3'）
DNA-1	Template:<br>AGCTAAAGCGCATCCCG
	Primer:<br>FAM-CGGGATGCGCTT
DNA-2	Top strand:<br>TTTTTTTTTTTTTTTTTTTTTTTTTTTTTTTTTTTCCCGGAACGAGTCAAGC<br>GCATCCCG
	Bottom strand:<br>FAM-CGGGATGCGCTTGACTCGTTGG
DNA-3	Top strand:<br>CCCGGAACGAGTCAAGCGCATCCCGTTTTTTTTTTTTTTTTTTTTTTTTTT<br>TTTTTTTTT
	Bottom strand:<br>FAM-CGGGATGCGCTTGACTCGTTGG
DNA-4	Template strand:<br>ATCCGGCTTGGTTGTTGCGTTAAGCGCATCCCGACGGAATGGCACGAAG<br>CTGTTGCTCA
	Primer strand:<br>FAM-CGGGATGCGCTT
DNA-5	Template strand:<br>ATCCGGCTTGGTTGTTGCGTTAAGCGCATCCCGACGGAATGGCACGAAG<br>CTGTTGCTCA
	Complementary strand:<br>TGAGCAACAGCTTCGTGCCATTCCGTCGGGATGCGCTTTTTTTTTTTTTT<br>TTTTTTTTT
	Primer strand:<br>FAM-CGGGATGCGCTT
